# Synthesis and Consecutive Reactions of α-Azido Ketones: A Review

**DOI:** 10.3390/molecules200814699

**Published:** 2015-08-13

**Authors:** Sadia Faiz, Ameer Fawad Zahoor, Nasir Rasool, Muhammad Yousaf, Asim Mansha, Muhammad Zia-Ul-Haq, Hawa Z. E. Jaafar

**Affiliations:** 1 Department of Chemistry, Government College University Faisalabad, Faisalabad-38000, Pakistan, E-Mails: sadia.faiz01@gmail.com (S.F.); nasirrasool@gcuf.edu.pk (N.R.); dryousafsmor@gmail.com (M.Y.); mansha.asim@gmail.com (A.M.); 2 Office of Research, Innovation and Commercialization, Lahore College for Women University, Lahore-54600, Pakistan; E-Mail: ahirzia@gmail.com; 3 Department of Crop Science, Faculty of Agriculture, Universiti Putra Malaysia, Serdang-43400, Selangor, Malaysia

**Keywords:** α-azido ketones, synthetic applications, heterocycles, click reactions, drugs, azides

## Abstract

This review paper covers the major synthetic approaches attempted towards the synthesis of α-azido ketones, as well as the synthetic applications/consecutive reactions of α-azido ketones.

## 1. Introduction

α-Azido ketones are very versatile and valuable synthetic intermediates, known for their wide variety of applications, such as in amine, imine, oxazole, pyrazole, triazole, pyrimidine, pyrazine, and amide alkaloid formation, *etc.* α-Azido ketones have been employed for the synthesis of a number of biologically important heterocyclic compounds. α-Azido ketones produce synthetically useful intermediates such as α-amino ketones, α-azido-β-hydroxy ketones, and β-amino alcohols [1].

**Figure 1:**
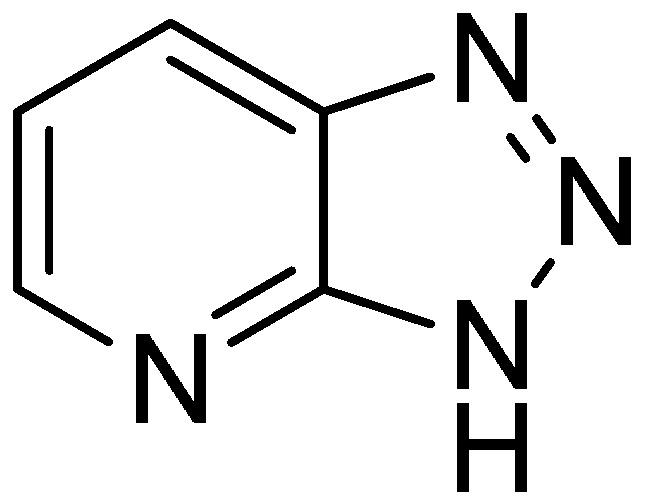
Pyridinyl-1,2,3-triazoles.

α-Azido ketones react with terminal alkynes and afford 1,2,3-triazoles in moderate to good yields through copper(I) catalyzed alkyne-azide 1,3-cycloaddition reaction (CuAAC, Sharpless-Meldal reaction). These molecules display valuable pharmaceutically important properties such as antiallergic, antihistamine, antibacterial effects, tuberculosis inhibition, anti-HIV, antitumor activities and agrochemical applications. An example of these triazoles is pyridinyl-1,2,3-triazole (Figure 1) which inhibits TGF-β-induced transcriptional activation of ALK signaling. One such compound is under preclinical tests [2].

**Figure 2:**
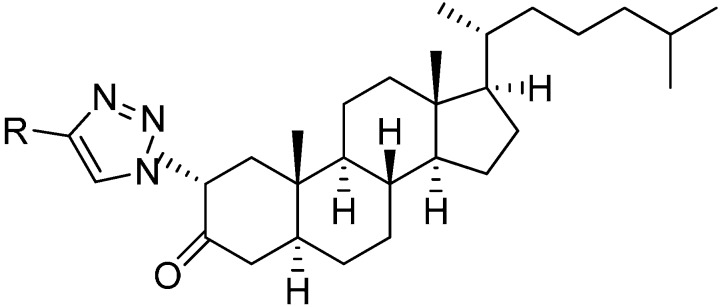
2α-Triazole-5α-cholestan-3-one.

1,2,3-Triazoles obtained from α-azido ketones have high chemical stability. They also contain strong dipole moment and an aromatic character with good hydrogen bond accepting ability. Various triazolyl ketone derivatives of cholesterol obtained by CuAAC reaction with various cholesterol derivatives show very good anti-proliferative activities against three human cancer cell lines for example, 2α-triazole-5α-cholestan-3-one [3] (Figure 2).

**Figure 3:**
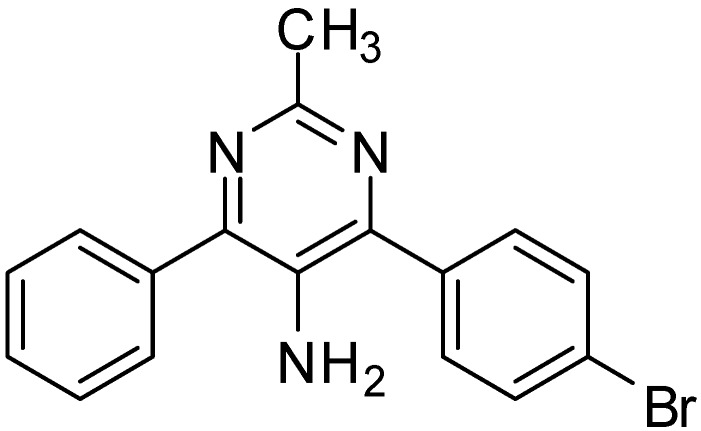
5-Aminopyrimidine derivative.

Recently, α-azido vinyl ketones have been used for the synthesis of pyrimidines, which possess antitumor, antiinflammatory, antiviral, antiproliferative activities, as well as they have the potential to inactivate human DNA repair. Another important derivative is 5-aminopyrimidine (Figure 3) which exhibits various important pharmacological activities such as antianoxic and antilipid peroxidation to ameliorate brain ischemic damage [4].

**Figure 4:**
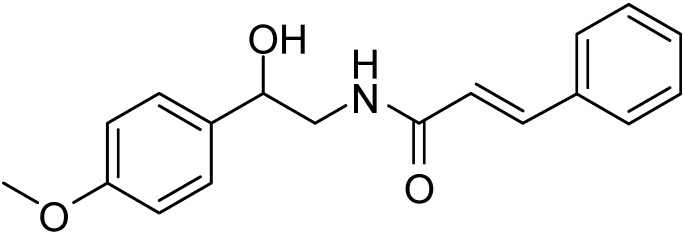
Aegeline.

α-Azido ketones have been used as useful precursors in the synthesis of various alkaloid amides, which are used for metabolic diseases, for example; aegeline (Figure 4). Aegeline is known to be dual acting agent and exhibits antioxidant, antihyperlipidemic and antihyperglycemic activities [5].

**Figure 5:**
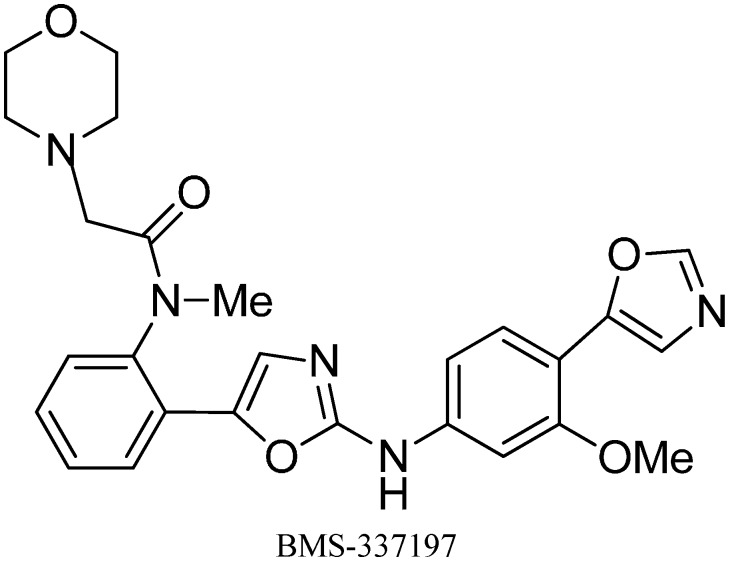
A novel IMPDH inhibitor.

Another application of α-azido ketones is in the synthesis of inhibitors of inosine monophosphate dehydrogenase (IMPDH) (Figure 5) which catalyzes the nicotinamide adenosine (NAD) dependent conversion of inosine 5ʹ-monophosphate (IMP) to xanthosine 5ʹ-monophosphate (XMP). IMPDH is an attractive target for immunosuppressive, anticancer, and antiviral therapies [6].

## 2. Literature Review

Since α-azido have been employed in the synthesis of biologically important heterocyclic compounds such as oxazoles, pyrroles and pyridines, as well as for the synthesis of synthetically useful intermediates such as α-amino ketones, α-azido-β-hydroxy ketones and β-amino alcohols, a schematic outline of the synthetic applications of α-azido ketone is given here.

**Scheme 1:**
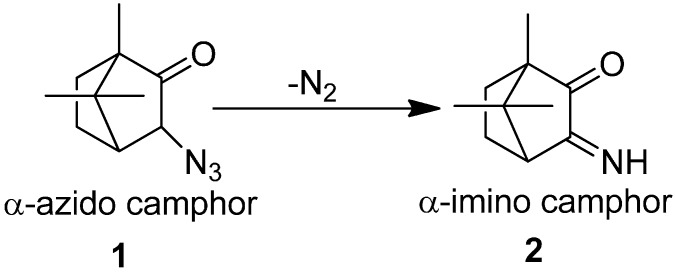
Synthesis of α-imino camphor **2**.

The azido group has received attraction since phenyl azide was discovered in 1864 and hydrogen azide was discovered in 1890. Due to their attractive aspects, a literature review describing the chemistry of organic azides was presented by Boyer, which explained the chemical properties and reactivity of alkyl and aryl azides. Forster reported in 1905 the acid and base catalyzed decomposition reactions of azido group [7]. Their investigation involved the study on the decomposition of α-azido ketones, aldehydes and esters. In this study, α-azido camphor **1** was converted to α-imino camphor **2** by the loss of nitrogen molecule (Scheme 1).

**Scheme 2:**
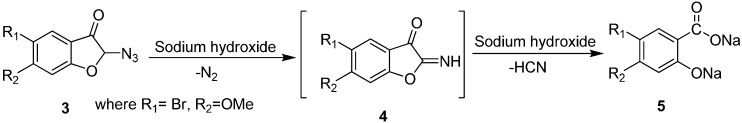
Preparation of sodium salt of 5-bromo-4-methoxysalicylic acid (**5**) from α-azido ketone **3**.

Similarly, Fries in 1926 converted 2-azido-5-bromo-2,3-dihydro-6-methoxy-3-coumaranone (**3**) into the sodium salt of 5-bromo-4-methoxysalicylic acid (**5**) [8]. This transformation was done by using NaOH via the formation of imine intermediate **4** and involved loss of N_2_ (Scheme 2).

**Scheme 3:**
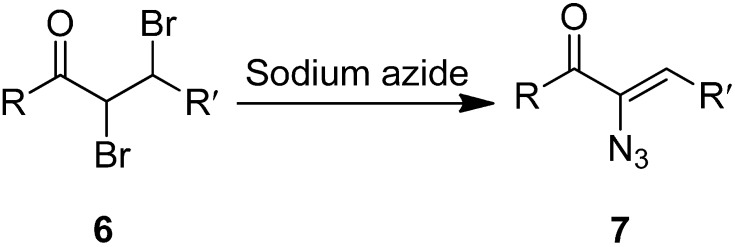
Conversion of α-azido ketone **6** to azido vinyl ketone **7**.

In another study, azido vinyl ketone **7** was synthesized from a dibromo ketone using sodium azide. The mechanism for the ketone formation was proposed by Cromwell in 1946 [9]. It involved the displacement of bromide ion from the α-carbon of **6** by azide ion through a S_N_2 reaction followed by elimination of HBr. The bromide ion was displaced from the α-carbon in **6** since the α-carbon in the former was activated by the carbonyl group (Scheme 3).

**Scheme 4:**
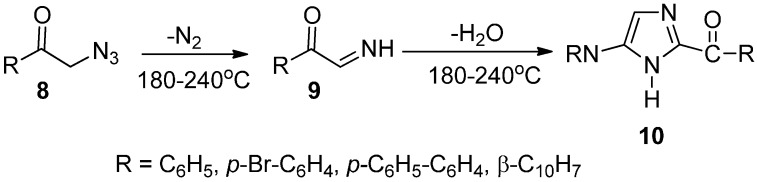
Preparation of imidazole derivatives **10**.

As an extension of the exploration of the preparation of heterocyclic compounds, the synthesis and properties of phenacyl azide were investigated. An efficient synthetic approach for the synthesis of some substituted imidazole derivatives from phenacyl azides and structurally related α-azido ketones was developed by Boyer in 1952 [10]. In this approach, phenacyl azide **8** and structurally related α-azido ketone led to the loss of nitrogen when these compounds were heated in the temperature range between 180 °C and 240 °C in an inert solvent. As a result, the α-imino ketone intermediate **9** was generated, which underwent dimerization and dehydration to afford good yields of imidazole derivatives **10** (Scheme 4).

**Scheme 5:**
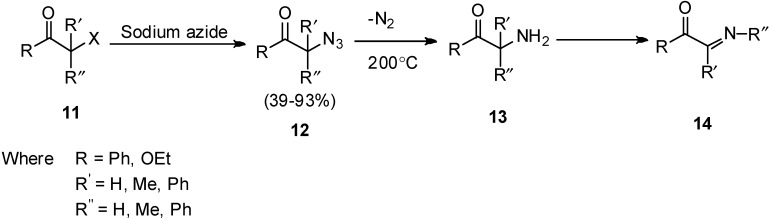
Pyrolysis of α-azido ketone **12** to α-imino carbonyl compounds **14**.

In 1952, the pyrolysis of α-azido carbonyl compunds was performed by Boyer [11] which resulted in the formation of α-imino carbonyl compounds. It was demonstrated that pyrolysis occured at moderate rate at 200–220 °C. In this report, it was observed that pyrolysis of azide occured in the presence of *o*-phenylene diamine which after thermal decomposition of azido group followed by rearrangement resulted in the formation of imine derivatives **14** (Scheme 5).

**Scheme 6:**
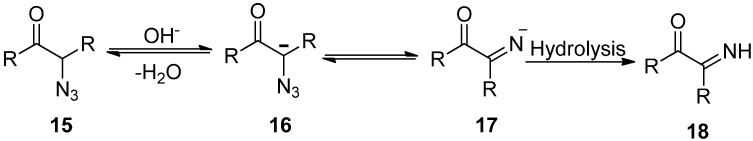
Base catalysed transformation of α-azido ketones **15** to imines **18**.

It was shown that imines could easily be converted to the corresponding dicarbonyl compounds upon hydrolysis. The cases in which α-carbon was tertiary, the compound was inert towards decomposition. For example, the formation of carbanions due to the removal of protons by the base from α-azido ketones was reviewed by Boyer in 1953. This carbanion was then converted into imine **18** after losing a N_2_ molecule, followed by reaction with water (Scheme 6).

**Scheme 7:**
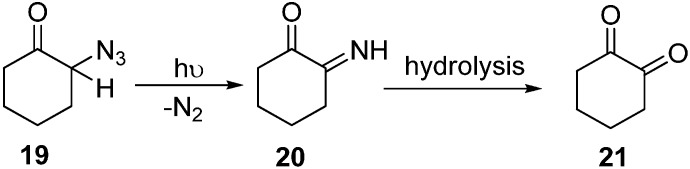
Preparation of α-diketone **21** from α-azido ketone **19**.

Earlier, a study of the photochemical decomposition of these compounds was carried out, but later on, many factors brought attention towards their acid-base catalyzed reactions. Keeping those considerations in mind, some reactions of alicyclic α-azido ketones were studied by Edward in 1964 [12]. 2-Azido ketone **19** was prepared in good yield in the form of yellow oil which gave an intense absorption at 2110 cm^−1^ by the reaction of 2-chlorocyclohexanone with sodium azide in DMSO, However, it could not be distilled under reduced pressure without considerable decomposition and polymerization. When a hexane solution of 2-azidocyclohexanone was irradiated in a quartz vessel with ultravoilet light, liberation of nitrogen gas resulted. This and other material left in the hexane solution showed absorption bands at 3400 cm^−1^ and 1670 cm^−1^ which are characteristic of α-imino ketones **20**. The product could only partially be distilled under 15 mm pressure (Scheme 7).

The sensitive imino ketone could rapidly be hydrolyzed to the α-diketone which proved to be a smooth reaction and resulted in good yields of 1,2-cyclohexanedione (**21**).

**Scheme 8:**
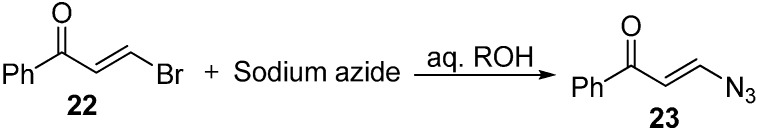
Synthesis of β-azidovinyl ketone **23**.

Later, an efficient method for the synthesis of β-azidovinyl ketone **23** from β-chloro-(or bromo-) vinyl ketones **22** was reported by Rybinskaya [13]. However, this nucleophilic substitution proceeded through retention of configuration around the C=C bond. In this reaction, conjugate addition of azide ion to the carbon carrying the Br group, of the α,β-unsaturated ketone was performed. It was also observed that when a similar procedure is perfomed on the regioisomeric α-bromovinyl ketone, addition of N_3_^−^ ion is observed rather than substitution (Scheme 8).

Base promoted reactions of α-azido ketones with aldehydes and ketones, either result in α-azido-β-hydroxy ketones or 2,5-dihydro-5-hydroxyoxazoles, among which the former are important 1,2,3-trifunctionalized synthons.

**Scheme 9:**
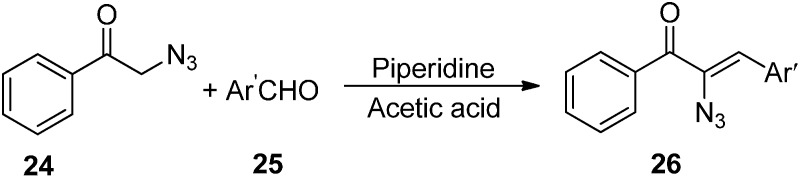
Synthesis of vinyl azido ketones **26** from phenacyl azide **24**.

Since α-azido ketones have an α-hydrogen, they are highly base sensitive. It was observed that deprotonation of α-azido ketones generated an anion which after undergoing loss of a nitrogen molecule was converted into an imino anion (Scheme 8). In our knowledge, only two published reports have shown that either of these anions could be used as electrophiles. Knittel [14] in 1970 reported the condensation of phenacylazide (**24**) and aromatic aldehydes **25** catalyzed by piperidinium acetate (Scheme 9). The reaction occured between the enol of the azido ketone and the iminium ion of aldehydes so neither β-hydroxy-α-azido ketone nor its intermediate was formed and rather vinyl azido ketones **26** were obtained.

**Scheme 10:**
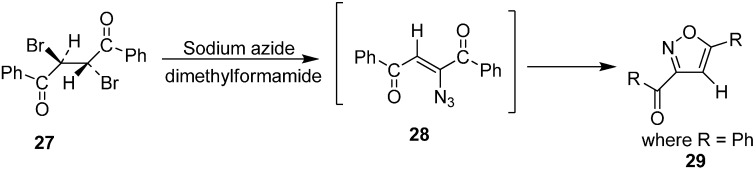
Conversion of *meso*-1,2-dibenzoylethylene **27** to isoxazole **29**.

Further, studies on the geometrical configuration of vinyl azide products was carried out by Hassner in 1971. As it is known both *cis* and *trans* α-bromo vinyl ketones could be transformed into α-azido vinyl ketones but the *trans* isomer was assumed to be thermodynamically more stable [15]. In a reaction of *meso*-1,2-dibenzoylethylene (**27**) with two equiv of NaN_3_, 3-benzoyl-5-phenyl isoxazole (**29**) was obtained. The formation of this product occurred due to the decomposition of vinyl azide **28**, which has both α-azido- and β-carbonyl groups in a *cis*-configuration. This finding was in agreement with the result of Nesmeyanov and Rybinskaya that *trans*-15 could be separated in pure form but *cis*-15 was unstable (Scheme 10).

**Scheme 11:**
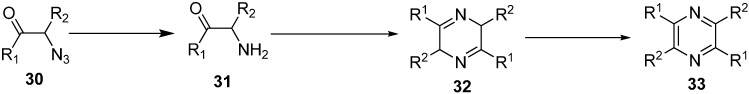
Synthesis of pyrazine derivatives **33**.

α-Azido ketones have also been used for the synthesis of 1,2-amino alcohols or α-amino ketones by carrying out the reduction of corresponding α-azido ketone. In this case, either the carbonyl or azide group undergo chemoselective reduction. By using this protocol, in 1980 Nakajima [16] studied the catalytic reduction of different phenacyl azides and aliphatic α-azido ketones **30** to form α-amino ketone **31** which, after reacting over Pd/C in ethanol in the presence of acetic acid, formed pyrazine **33**. In other cases, an intermediate dihydropyrazine **32** was also formed, which was oxidized to pyrazine (Scheme 11).

**Scheme 12:**
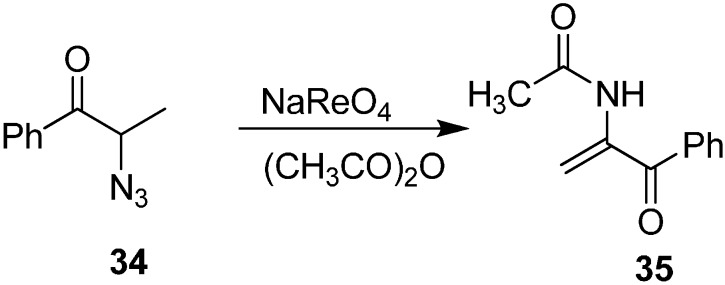
Formation of 2-(acetylamino)-2-alken-1-ones **35**.

A catalytic amount of perrhenate can be used for the thermally induced removal of nitrogen from both cyclic and acyclic α-azido ketones. Hence, decomposition of 2-azido ketones **34** to 2-(acetylamino)-2-alken-1-ones **35** by perrhenate catalyst was done by Effenberger in 1985 [17]. The products were formed in good yield when acetic anhydride was used (Scheme 12).

**Scheme 13:**
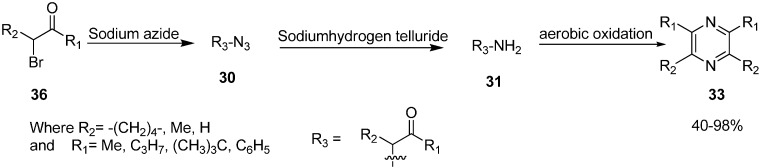
Conversion of α-azido ketones to pyrazines **33**.

α-Azido ketones are easily converted into pyrazines as established by Suzuki *et al.* in 1986 [18]. They demonstrated the formation of pyrazine via self condensation of α-amino ketones, when α-azido ketones were treated with sodium hydrogen telluride in ethanol at room temperature (Scheme 13).

**Scheme 14:**
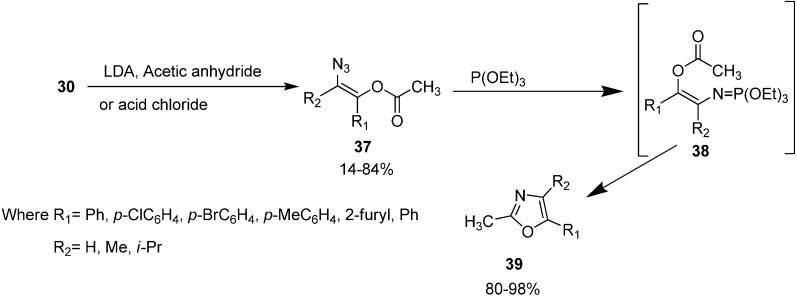
Synthesis of oxazole derivative **39** from α-azido ketones **30**.

Recently, oxazoles have seen revitalizing importance due to their utility as synthetic intermediates. These are of considerable interest due to their presence in naturally occurring products such as alkaloids (texamine, texaline, *etc*.) and macrocyclic antibiotics. In many previous oxazole syntheses, strong dehydrating agents such as (P_2_O_5_, H_2_SO_4,_ SOCl_2_, *etc.*) or Lewis acid were usually required but now instead of acidic reagents, P(OEt)_3_ is being used and as a result furyl- or pyridyl- substituted oxazole derivatives are easily produced. The synthesis of oxazole by *N*-acylamino phosphonium salts involving the starting α-azido ketones, triphenylphosphine and acyl halides was reported by Zbiral and coworkers and was called type A synthesis. The recently used intramolecular aza-Wittig approach for the synthesis of oxazoles from α-azido ketones, based on *N*-*C*-*C*-*O*-*C* atomic unit for ring construction, was reported by Takeuchi in 1989 [19] who called it the B type approach. In their approach, initially α-azido ketones were prepared from α-bromo ketones. Then α-azido ketone **30** was converted to (*Z*)-β-(acyloxy)vinyl azide **37** by using selective enol acylation and the resulting (*Z*)-β-(acyloxy) vinyl azide was reacted with triethyl phosphite to form the oxazole derivative **39** by a Staudinger reaction, followed by an intramolecular aza-Wittig reaction (Scheme 14).

Both A and B type approaches are useful for the preparation of oxazoles under mild, non-acidic conditions but yields seem to be better when the B type approach is employed mainly due to the fewer side reactions.

**Scheme 15:**
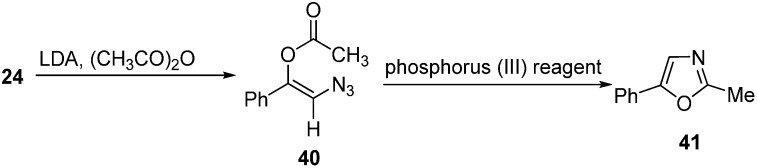
Formation of isoxazole **41**.

Takeuchi also reported the formation of lithium enolates of phenacyl azides from α-azido ketones **24** at −78 °C. It was suggested that this anion could be captured with acetyl chloride/acetic anhydride to synthesize *O*-acylated vinyl azides **40**, followed by reaction with phosphorus (III) reagents to form oxazole derivatives **41** (Scheme 15).

**Scheme 16:**
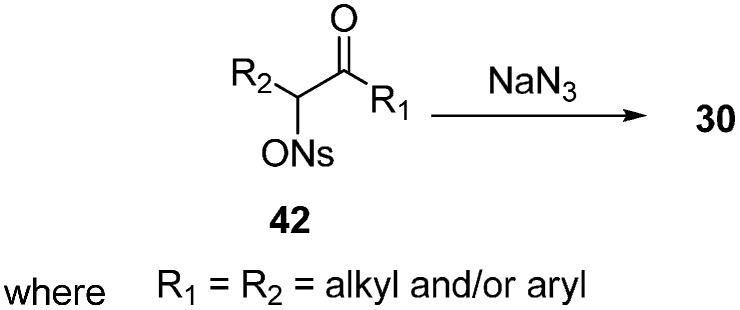
Preparation of α-azido ketones **30** from α-nosyloxy ketones **42**.

Traditionally α-azido ketones **30** have been synthesized by the nucleophilic substitution of α-halo ketones. This procedure gives excellent yields for phenacyl halides but it does not work well for substrates having β-hydrogens or conjugating groups at the β-carbon. The synthesis of unsymmetrical α-azido ketones could be achieved only if the starting α-halo ketones are prepared regiospecifically. In order to achieve a useful pathway towards α-azido ketones, Patonay in 1994 [20] reviewed the synthesis of α-azido ketones from α-((4-nitrobenzene)sulfonyl)oxy ketones (α-nosyloxy ketones) **42** by nucleophilic substitution of azide in different solvents (Scheme 16). Here, great yields of **30** were obtained in all of the solvents used, however the time period of the reaction varied because the solubility of sodium azide in these solvents was different at large scale. By the use of sodium azide in acetone at room temperature, a variety of α-nosyloxy ketones were transformed into the corresponding α-azido ketones. The reaction gave excellent yields for compounds having diverse structures. The single factor which could lead to a lesser yield was the volatility of the azide or the product uncertainity. The importance of this method lies in the fact that it was carried out under moderate reaction conditions which gave high yield of base sensitive products.

**Scheme 17:**
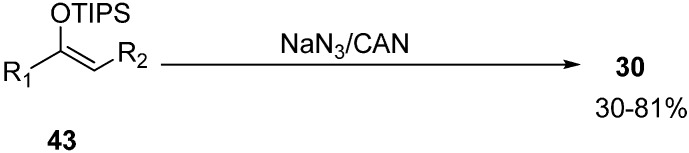
Synthesis of α-azido ketones **30**.

Another method for the synthesis of α-azido ketones was proposed by Magnus in 1995 which involved oxidative azidation of triisopropylsilyl enol ethers for the direct synthesis of α-azido ketones rather than by normal nucleophilic substitution of halogen ion with azide anion [21] (Scheme 17).

A wide variety of triisopropylsilyl enol ether derivatives underwent oxidative azidation and formed α-azido ketones in average to good yields. The reaction is not stereospecific as mixtures of diastereomers were obtained. The methodology also involved the introduction of azide at a tertiary carbon and 2-azido-2-methylcyclohexanone was produced as an example.

**Scheme 18:**
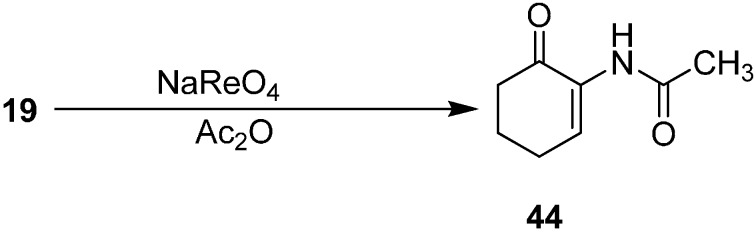
Preparation of acyl enamine derivative **44** from α-azidoketone **19**.

After achieving the oxidative azidation of triisopropylsilyl enol ethers, attention was turned to the transformation of the azide to an acyl enamine derivative. For example, 2-(acetylamino)alk-2-ene-1-ones **44** were prepared from α-azido ketone **19** in acetic anhydride with a catalytic amount of triflic acid (1 mol %), HCl (1 mol %) and sodium perrhenate (1 mol %) (Scheme 18).

The decomposition of azide with sodium perrhenate also occured in the presence of methyl chloroformate. For example, α-azido cycloalkanone derivatives were employed to yield the corresponding 2-amino(methoxycarbonyl)cycloalk-2-ene-1-ones in 16%–56% yield.

**Scheme 19:**
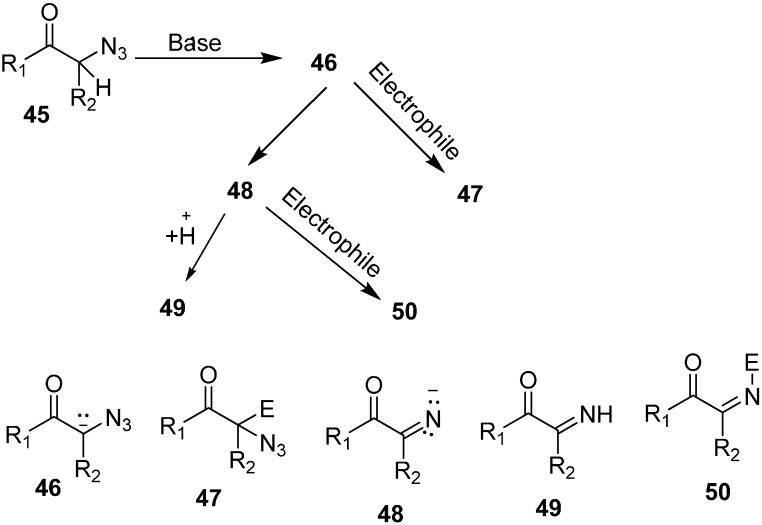
Base promoted conversion of **45** to products **47**, **49** and **50**.

In their study, the base-promoted conversion of α-azido ketones was also carried out when aldehydes and ketones were used as electrophiles. In this regard, the formation and trapping of different anions with carbon electrophiles was studied by Patonay in 1995 [22]. The formation of product depended upon the type of anion trapped during the reaction, e.g., α-azido-β-hydroxy ketones were formed when an aldol reaction occured between an aldehyde and an α-azido ketone enolate whereas 2,5-dihydro-5-hydroxy oxazoles were synthesized when an imino ion formed by nitrogen loss from an enolate of α-azido ketone was trapped (Scheme 19).

**Scheme 20:**
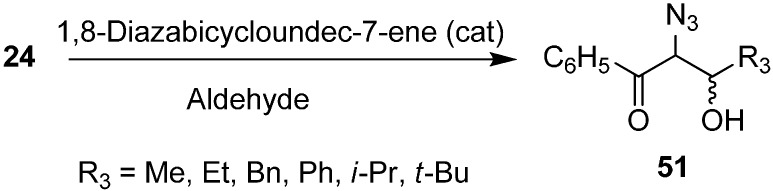
DBU promoted conversion of **24** to α-azido-β-hydroxyketones **51**.

The synthesis of α-azido-β-hydroxy ketones by reacting phenacyl azide with aldehydes in the presence of 1,8-diazabicyclo[5.4.0]undec-7-ene (DBU) was also reported by Patonay in 1995 (Scheme 20).

**Scheme 21:**
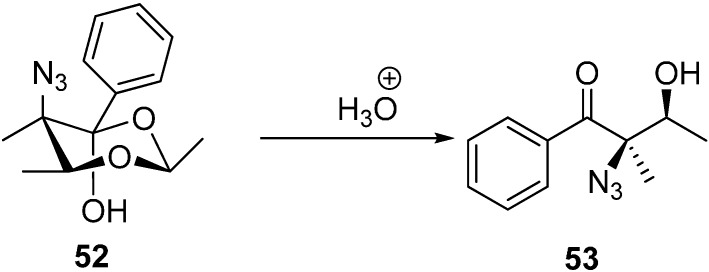
Formation of (2*R*,3S)-2-azido-3-hydroxy-2-methyl-1-phenylbutan-1-one (**53**).

The same research group reported the synthesis of (2*R*,3S)-2-azido-3-hydroxy-2-methyl-1-phenylbutan-1-one (**53**) by the acidic hydrolysis of (*2S*,5*R*,6*S*)-5-azido-2,5,6-trimethyl-4-phenyl-1,3-dioxan-4-ol (**52**) (Scheme 21).

**Scheme 22:**
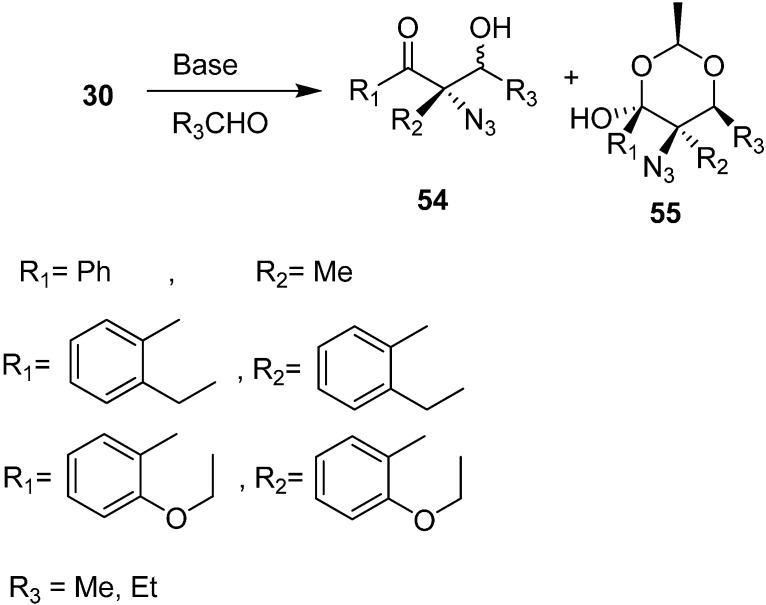
Preparation of aldol products **54** and 1,3-dioxane derivatives **55** from α-azido ketones.

They extended the scope of their methodology towards the synthesis of aldol products **54** and 1,3-dioxane derivatives **55** by the reaction of α-azido ketones with aldehydes in the presence of 1 equiv of DBU (Scheme 22).

**Scheme 23:**
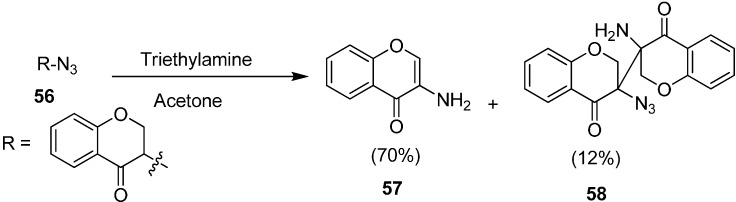
Synthesis of products **57** and **58** from 3-azidochroman-4-one (**56**).

The synthesis of 3-amino-4*H*-chromen-4-one and 3-amino-3ʹ-azido-[3,3ʹ-bichroman]-4,4ʹ-dione (**58**) by the reaction of 3-azidochroman-4-one (**56**) with triethylamine (TEA) in the presence of acetone was reported by the same group (Scheme 23).

**Scheme 24:**
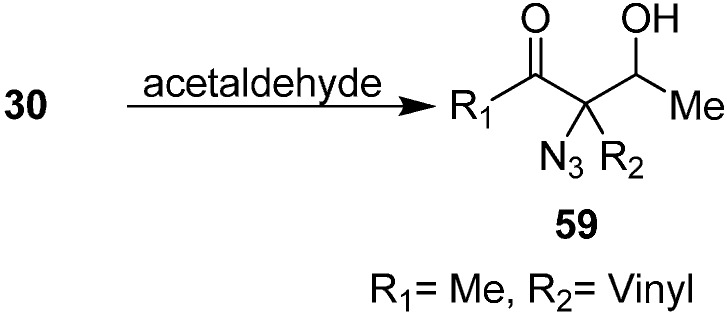
Preparation of aldol products **59** from α-azido ketones.

Patonay reviewed the synthesis of aldol product 3-azido-3-(1-hydroxyethyl) hex-5-en-2-ones **59** by reacting 3-azidohex-5-en-2-ones **30** with acetaldehyde (Scheme 24).

**Scheme 25:**
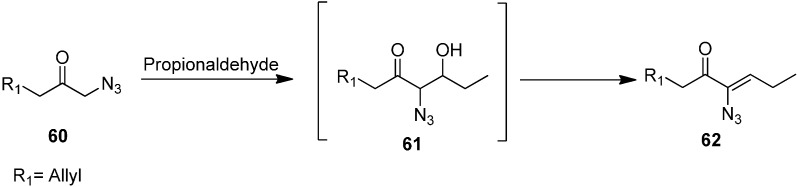
Synthesis of (*Z*)-6-azidonona-1,6-dien-5-one **62** from α-azido ketone **60**.

The aldol product (*Z*)-6-azidonona-1,6-dien-5-one **62** was synthesized by reacting 1-azidohex-5-en-2-one **60** with CH_3_CH_2_CHO (Scheme 25).

**Scheme 26:**
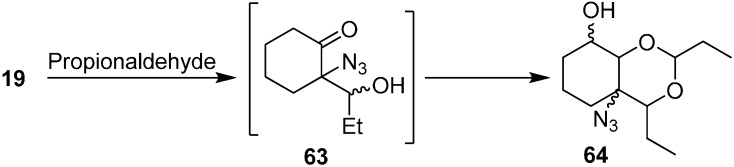
Preparation of aldol product **64** from α-azido ketone **19**.

**Scheme 27:**
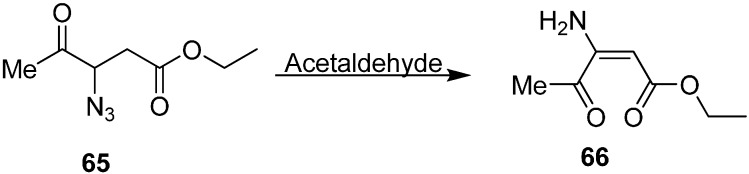
Preparation of (*Z*)-ethyl 3-amino-4-oxopent-2-enoate (**66**).

Similarly, the synthesis of aldol product azido-2,4-diethylhexahydro-4*H*-benzo[*d*][1,3]dioxin-8-ol (**64**) was obtained by reacting 2-azidocyclohexanone (**19**) with EtCHO (Scheme 26).

(*Z*)-ethyl 3-amino-4-oxopent-2-enoate (**66**) was also synthesized by reacting ethyl 3-azido-4-oxopentanoate (**65**) with MeCHO (Scheme 27).

**Scheme 28:**
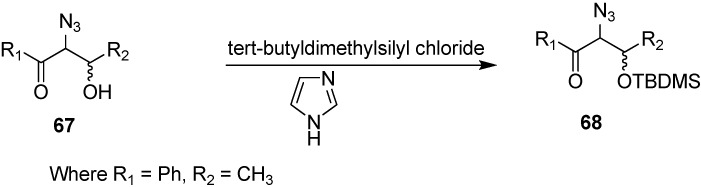
Synthesis of 2-azido-3-(*tert*-butyldimethylsilyloxy)-1-phenylbutan-1-one (**68**).

Patonay also performed the synthesis of 2-azido-3-(*tert*-butyldimethylsilyloxy)-1-phenylbutan-1-one (**68**) by reacting 2-azido-3-hydroxy-1-phenylbutan-1-one (**67**) with *tert*-butyldimethylsilyl chloride (TBDMSCl) in the presence of imidazole and DMF (Scheme 28).

**Scheme 29:**
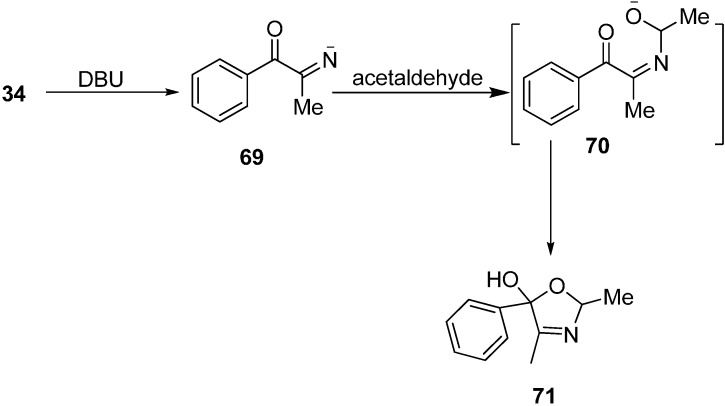
Synthesis of 2,4-dimethyl-5-phenyl-2,5-dihydrooxazol-5-ol (**71**) from **34**.

2,4-Dimethyl-5-phenyl-2,5-dihydrooxazol-5-ol (**71**) was obtained when 2-azido-1-phenylpropan-1-one (**34**) was reacted with MeCHO in DBU with the loss of N_2_ (Scheme 29).

**Scheme 30:**
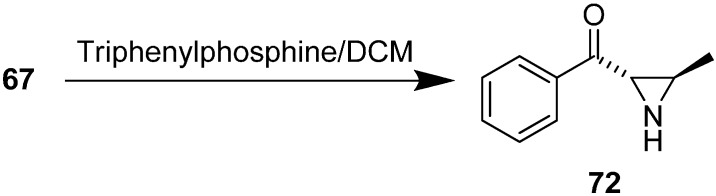
Synthesis of aziridine **72** from aldol product **67**.

The synthesis of ((2*S*,3*R*)-3-methylaziridin-2-yl) (phenyl) methanone **72** from 2-azido-3-hydroxy-1-phenylbutan-1-one **67** in the presence of Ph_3_P/CH_2_Cl_2_ was also illustrated (Scheme 30).

**Scheme 31:**
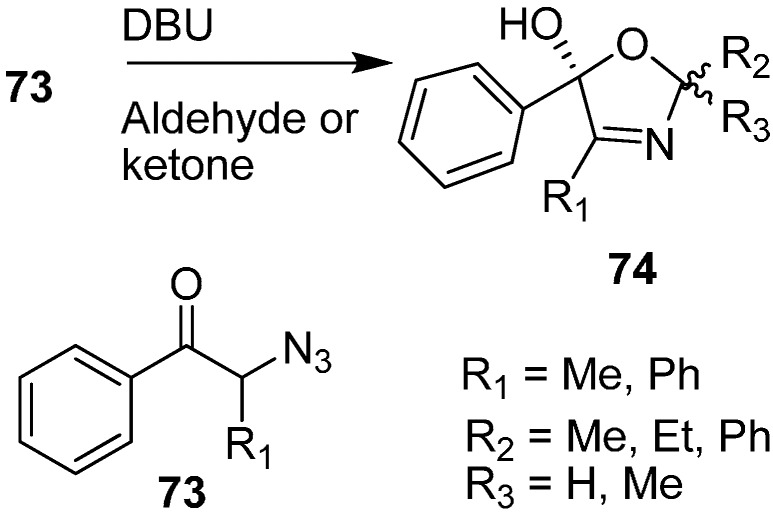
Preparation of oxazoline derivatives **74** from α-azido ketones **73**.

Moreover, the synthesis of oxazoline derivatives was achieved by reacting α-azido ketones with various aldehydes and ketones in the presence of DBU (Scheme 31).

In addition to organic azides, α-diazo carbonyl compounds are also used in the synthesis of organic heterocyclic and carbocyclic rings which are linked to natural products as well as for the synthesis of pharmacologically active compounds.

**Scheme 32:**
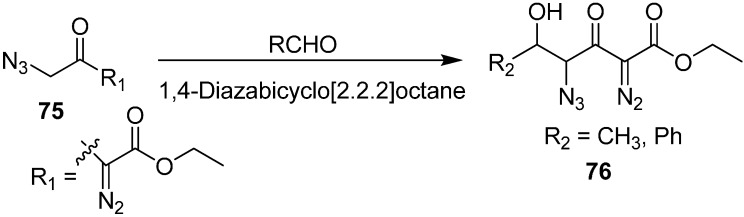
Synthesis of γ-azido-α-diazo-δ-hydroxy-β-keto esters **76** from azido diazo esters **75**.

The azido and diazo groups, being isoelectronic in nature and reactivity, are related to some extent, allowing azides to be thought of as diazo groups linked to a nitrogen atom. Recently, the synthesis of γ-azido-α-diazo-δ-hydroxy-β-keto esters **76** through an aldol type reaction of the azido diazo esters **75** with aldehydes catalyzed by DABCO was reported by Padwan in 1997 [23]. This condensation reaction was carried out under mild reaction conditions and resulted in high yields (Scheme 32).

**Scheme 33:**
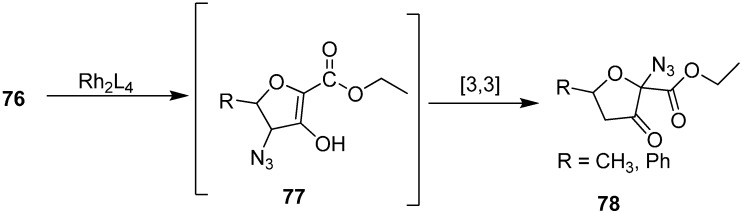
Preparation of 2-azido-3-furanones **78**.

Padwa and co-workers [24,25] also reported the conversion of γ-azido-α-diazo-δ-hydroxy-β-keto esters **76** into 2-azido-3-furanones **78**. The reaction was a rhodium (II) catalyzed intramolecular OH-insertion followed by [3+3] sigmatropic rearrangement of the transient allylic azide (Scheme 33).

Immobilized organocations that include quaternary ammonium ions are present in many triphase catalysts. Many useful and functionalized organic polymers and mineral supports are being used as triphase catalysts. Although by using triphase catalysts, advantages associated with the recovery of catalysts are seem, this system has some disadvantages associated with it. The limitations associated with triphase catalysts based on polymers include diffusion limitations, high cost, tendency to swell and reaction instability. To avoid these limitations, inorganic-based catalysts, e.g., oxides of metals, clays and zeolites are used instead of polymer-based catalysts, but these also suffer from disadvantages similar to those associated with polymer-based catalyst.

**Scheme 34:**
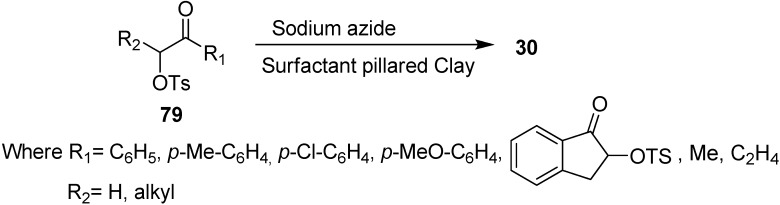
Preparation of α-azido ketones **30** from α-tosyloxy ketones **79** using pillared clays.

Due to these drawbacks associated with phase transfer catalysts, there was a need to develop new materials for the improvement of triphase catalysis. For this purpose, the immobilized onium cations pillared clays were used as catalysts in nucleophilic substitution reactions, for example, Varma in 1998 [26] developed the use of surfactant pillared clay for the synthesis of α-azido ketones **30** from easily available α-tosyloxy ketones **79** and NaN_3_. Their methodology proved to be a systematic procedure for the nucleophilic substitution of α-tosyloxy ketones with NaN_3_ and mild phase transfer catalysis conditions were used (Scheme 34).

Their study involved the screening of a wide range of solvents and chloroform was found to be the most suitable for this nucleophilic displacement which resulted in excellent yields and short reaction times. The methodology was applied to a variety of α-tosyloxy substrates such as aryl, cyclic and allylic ketones and reaction rates were found to be completely unaffected by the presence of electron withdrawing and donating substituents in the the α-tosyloxy ketones. However, the reaction rate was slower in the case of cyclic and allylic ketones. The reaction was also slow if carried out at room temperature and did not occur in the absence of catalyst. Moreover, the increase or decrease in the amount of organic clay did not affect the rate of reaction.

Recently, great interest has developed on the applicability of azides in the synthesis of organic compounds which could be used taking advantage of the broad spectrum of reactivity of these groups. Some useful reactions of azides for the formation of heterocycles are reductive cyclization, the Staudinger reaction, Curtius rearrangements, Schmidt rearrangements, nitrene insertions and radical cyclization. The cycloaddition of azides across double bonds gives a useful synthetic route towards triazolines, aziridines and other heterocycles. The vinyl azides are very much reactive and are available for photolysis, pyrolysis, and cycloaddition and can easily be attacked by nucleophiles as well as by electrophiles.

A synthetic strategy for the formation of nitrogen heterocycles by Vilsmeier cyclization of azides was developed by Majo in 1998 [27] which consisted of two steps: (i) the formation of an iminium salt from a Vilsmeier-active functional group and (ii) the intramolecular cyclization of an azide with the iminium salt followed by elimination of nitrogen.

**Scheme 35:**
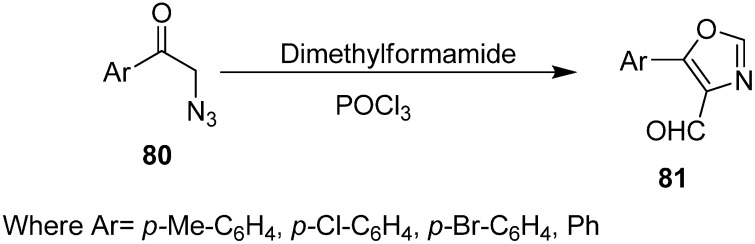
Vilsmeier cyclization of 2-azido acetophenones **80** to oxazoles **81**.

Due to the capacity of Vilsmeier reagents to form a wide variety of iminium species, a great scope is provided for intramolecular cyclization of azides with iminium species under Vilsmeier conditions. A number of alkene derivatives, carbonyl compounds, activated methyl and methylene groups, oxygen and nitrogen nucleophiles undergo reactions with Vilsmeier reagent to synthesize the corresponding iminium salts which upon cyclization under Vilsmeier conditions give a useful synthetic access to large numbers of heterocycles. For example, Vilsmeier cyclization of 2-azido acetophenones **80** provided an effective route for the synthesis of 5-aryloxazole-4-carboxaldehydes **81**. The reaction took place at 80–90 °C for 2–3 h by using three equiv. of Vilsmeier reagent and resulted in the formation of oxazoles in 36%–45% yield (Scheme 35). The azido acetophenone required for the synthesis of oxazole were prepared in good yield from 2-bromoacetophenone in the presence of DMF and POCl_3_ by using two equiv. of NaN_3_ at 15–20 °C for 20 min.

**Scheme 36:**
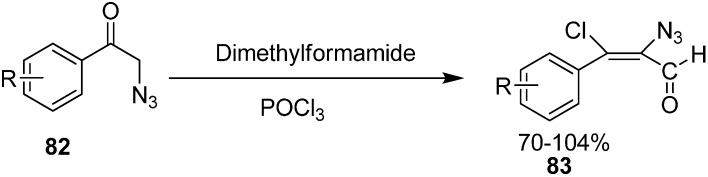
Preparation of α-azido-β-chlorovinyl aldehydes **83** from **82**.

Vattoly also reported the preparation in good yield of α-azido-β-chlorovinyl aldehydes **83** from 2-azidoacetophenones **82** by using six equiv. of Vilsmeier reagent (Scheme 36).

**Scheme 37:**
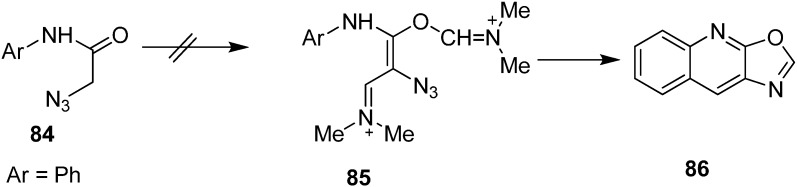
Syntheiss of oxazole-fused quinolines **86**.

Same research group also reported the synthesis of oxazole-fused quinolines **86** from *N*-aryl-2-azidoacetamides **84** by using Vilsmeier conditions (Scheme 37).

**Scheme 38:**
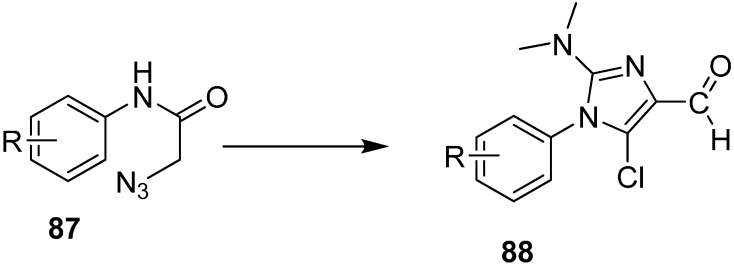
Preparation of imidazole derivatives **88**.

Similarly, the synthesis of Imidazoles **88** from 2-azidoacetanilides **87** by using Vilsmeier Reagent with low yield was also reported (Scheme 38).

**Scheme 39:**
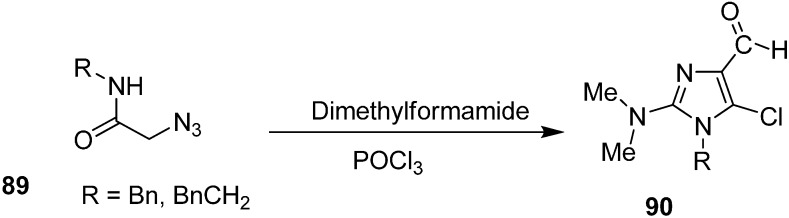
Synthesis of imidazole derivatives **90** from 2-azido-*N*-(phenylmethyl) acetamide **89**.

Vattoly *et al.* also reported synthesis of imidazoles **90** from 2-azido-*N*-(phenylmethyl) acetamide (**89**) by using Vilsmeier reagent (Scheme 39).

Little interest has been granted to the cathodic reduction of azides. The electroreduction of azides is carried out under protic conditions as well as under aprotic conditions. Based on the groups attached to the azide functionality, it can be converted either into an amine group with the loss of dinitrogen or undergo the elimination of azide anion.

**Scheme 40:**
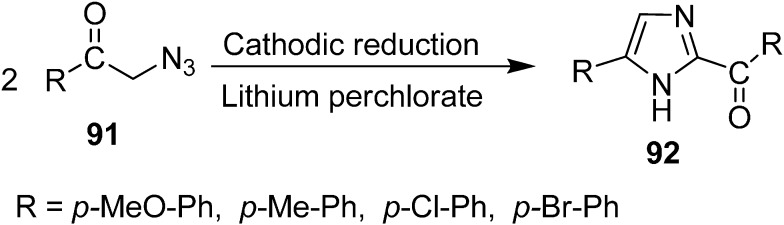
Synthesis of imidazoles **92** via electrochemical reduction of α-azido ketones.

The synthesis of 2-aroyl-4-arylimidazoles **92** in yields ranging from 70%–80% from α-azido ketones **91** by electrochemical reduction was performed by Batanero *et al.* in 1999 [28]. Phenacyl azides were prepared from phenacyl bromides and sodium azide and then reduced to 2-aroyl-4-arylimidazoles **92** by using DMF-LiClO_4_ at the mercury cathode at a controlled cathodic potential (Scheme 40).

**Scheme 41:**
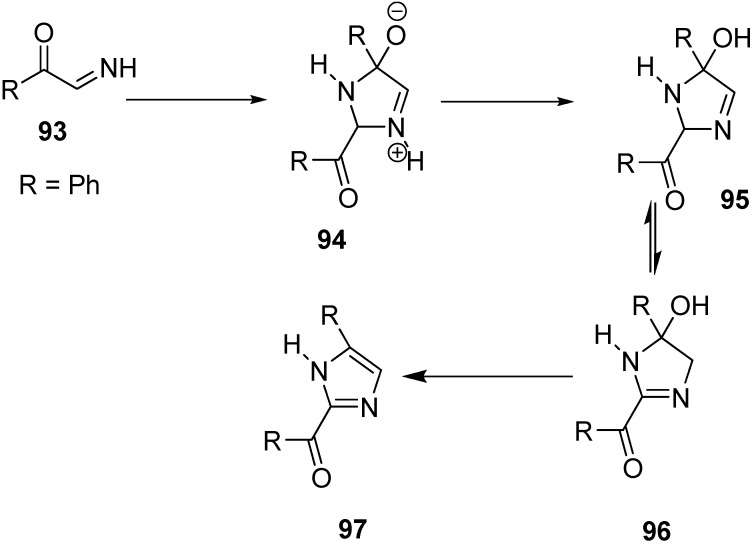
Synthesis of 2-benzoyl-4-phenylimidazole **97**.

A facile synthesis of 2-benzoyl-4-phenylimidazole (**97**) was carried out by Boyer and Straw through pyrolysis of phenacyl azide. In this case, phenylglyoxal imino derivative **93** was used as an intermediate. The enolate of acetophenone was generated by the reaction of acetophenone and sodium hydride followed by the treatment of the enolate with phenacyl azide in DMF. N_2_ gas was evolved and a check on the reaction medium confirmed the disappearance of the starting azide. The dimerization and dehydration are shown in the following Scheme 41.

**Scheme 42:**
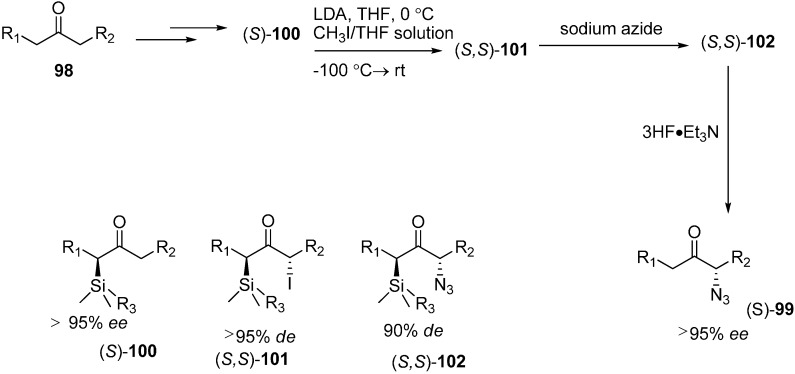
Enantioselective synthesis of α-azido ketones.

It is known that α-azido ketones are synthetically valuable sythons for protected and unprotected α-amino ketones. Although many methods have been proposed for the synthesis of α-azido ketones, none had been proposed for the asymmetric preparation of α-azido ketones until an enantioselective synthesis of α-azido ketone was accomplished by Enders *et al.* in 1999 [29]. In their strategy, replacement of the iodo group in α-silylated-iodo ketone **101** by NaN_3_ was the key step. This occured with great stereoselectivity. In the last step, a silicon bond breaking step took place with 3HF·Et_3_N for desilylation. They successfully generated α-silylated ketones in three or four steps by using SAMP/RAMP-hydrazone method (Scheme 42).

Sulfonyl azides are useful synthetic intermediates mainly used to carry out the transfer of diazo groups to the activated methylene of β-dicarbonyl compounds as well as to the poorly activated methylene of monocarbonyl compounds. In earlier times, toluene-4-sulfonyl (tosyl) azide (TsN_3_) was used, but recently the effectivity of the transformation of diazo groupw was markedly increased by the proper selection of sulfonyl diazo donors other than TsN_3_.

Among several powerful diazo donors, sulfonyl azides have a tendency to transfer the azido group. 4-Nitrobenzenesulfonyl azide (PNBSA) is used in the diazotization of cyclic ketones, while trifluoromethanesulfonyl (triflyl) azide behaves as powerful diazo donor to amines and enaminoketones.

In previous work, sulfonyl azide reactions of monocyclic and benzocyclic β-keto esters were studied. In the presence of triethylamine (TEA), these dicarbonyl compounds were transformed by TsN_3_ into attractive ring opened *N*-tosylcarbamoyl-substituted α-diazo esters. However, in the case of benzocyclic keto esters having conjugated aryl ketones, α-azidation occured.

**Scheme 43:**
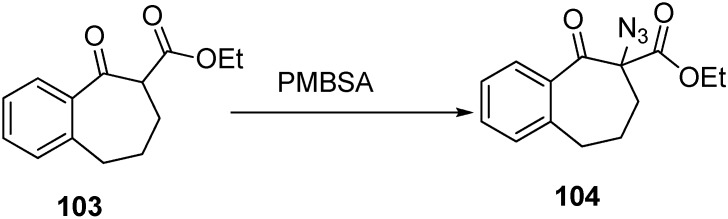
Synthesis of α-azido-β-keto ester derivative **104**.

Keeping in view these considerations, the synthesis of α-azido ketone derivative **104** was carried out by Benati *et al.* in 1999 [30] from benzosuberone **103**. They treated 4-methoxybenzenesulfonyl azide (PMBSA) with **103** in dry tetrahydrofuran (THF) in the presence of triethylamine (TEA) for 6 h and then after acidic work up, they separated **104** (Scheme 43).

**Scheme 44:**
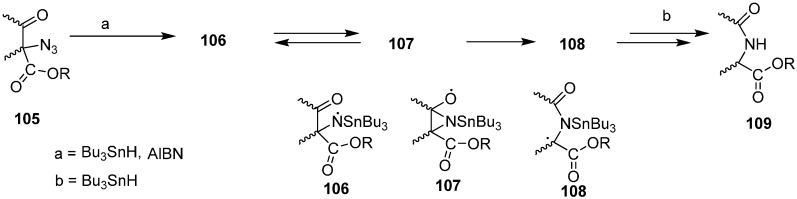
Preparation of alkoxycarbonyl-substituted amides and lactams **109**.

A number of acyclic and carbocyclic α-azido-β-keto esters can be synthesized from starting dicarbonyl compounds. Moreover, their radical chain reactions with tributyltin hydride have also been explored. These reactions result in the efficient preparation of alkoxycarbonyl-substituted amides and lactams and hence provide a useful route for regiospecific nitrogen addition to keto ester compounds. Hence through free radical nitrogen insertion reaction, Benati [31] reported in 1999 the synthesis of amides and lactams by the reaction of α-azido-β-keto esters **105** with tributyltin hydride (Bu_3_SnH) (Scheme 44).

**Scheme 45:**
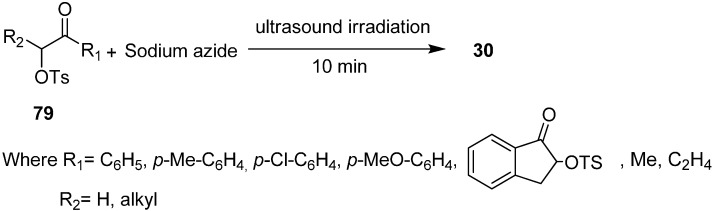
Synthesis of α-azido ketones from α-tosyloxy ketones **79**.

The Varma group [32] also reported in 1999 the synthesis of α-azido ketones **30** from α-tosyloxy ketones **79** and sodium azide under ultrasound irradiation or triphase catalysis conditions. They reported the comparison of use of surfactant pillared clay material and use of sonochemistry in nucleophilic substitution reactions (Scheme 45).

Significant acceleration in the rate of reaction was observed under ultrasound irradiation as compared to classical conditions (*i.e.*, under refluxing conditions). The reaction was further accelerated when a small quantity of surfactant pillared clay was added, which showed the activity enhancement of ultrasound on the triphase catalyst system. Hence the reaction was completed with sonication in the absence of pillared clay which showed that ultrasound could be used as a suitable alternative for phase transfer catalysts in nucleophilic substitution reactions.

**Scheme 46:**
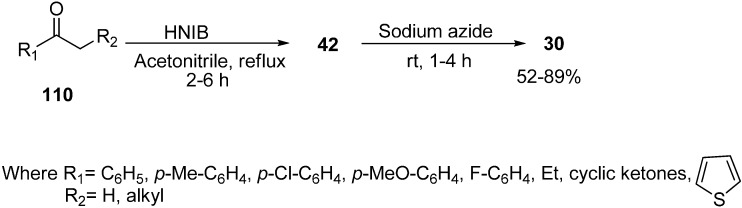
One-pot synthesis of α-azido ketones.

Recently it has been discovered that α-nosyloxy ketones, which also serve as precursors for the synthesis of α-azido ketones, could be synthesized by the reaction of ketones with [hydroxy(*p*-nitrobenzenesulfonyloxy)iodo]benzene (HNIB) in acetonitrile under refluxing conditions. However as a supplement of this method, a new and novel one-pot synthesis of α-azido ketones from their corresponding ketones was reported by Lee in 2000 [33]. In this approach, different ketones **110** were first reacted with HNIB in acetonitrile. The reaction was maintained at reflux for a time period of 2–6 h. This resulted in the formation of α-nosyloxy ketone intermediates **42** which then underwent nucleophilic substitution reaction with NaN_3_ (2.0 equiv.) at room temperature for the time period of 1–4 h and gave corresponding α-azido ketones **30** in high yields (Scheme 46).

It was observed that most of the ketones under examination were easily transformed to their corresponding α-azido ketones in a one-pot synthesis. However, yields were low in the case of cyclic ketones due to the loss of products under the work up conditions. All products showed spectral data consistent with the allocated structures. Addition of 18-crown-6 as a phase transfer reagent along with NaN_3_ caused no difference in reaction times and yield. Even replacing HNIB by Koser’s reagent, [hydroxyl(tosyloxy)iodo]benzene (HTIB), in the sulfonyloxylation step under same reaction conditions resulted in low yields.

β-Lactones are actually masked aldol products and thus are important starting precursors in natural and unnatural product synthesis. There are only few direct methods for the synthesis of these heterocyclic compounds in optically pure form. Recently, a number of direct procedures have been developed based on various cinchona alkaloids like quinidine and quinine which are useful catalysts for net [2+2] cycloaddition of carbonyl compounds such as chloral and ketene to synthesize β-lactones in excellent yield. However, these methods are of limited effectiveness due to the direct use of activated aldehyde and alkene generator. To start with, while addressing these limitations, such kind of reaction conditions were developed that could allow the use of ketene generated *in situ .* Triethylamine was used both as a base to effect dehydrochlorination as well as a nucleophile to facilitate the reaction between ketene and activated aldehydes. Keeping these considerations in mind, a modified procedure was developed in 2000 by Tennyson [34] for the preparation of a dichlorinated-β-lactone in optically pure form. This chlorinated β-lactone was transformed into a number of useful chiral precursors such as chloroepoxides, α-azido ketones, vinyl chlorides and propargylic benzyl ethers.

**Scheme 47:**
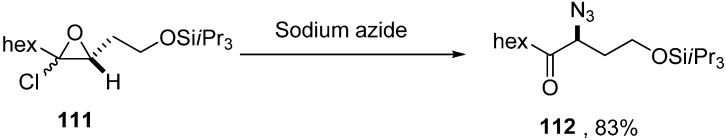
Synthesis of α-azido ketone **112**.

A number of synthesized β-lactones were converted to a chloroepoxides in good yield, first by reducing the β-lactones to diols in the presence of DIBAL-H, followed by treatment with NaH. This chloroepoxide **111** was then transformed into α-azido ketone **112** using NaN_3_ in aqueous DME. There was no loss in optical purity as determined by chiral phase HPLC (Scheme 47).

**Scheme 48:**
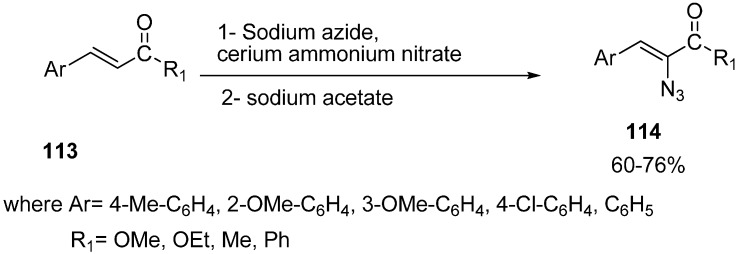
Preparation of α-azido-α,β-unsaturated ketones **114**.

Nair reported in 2000 the cerium (IV) ammonium nitrate assisted addition of azide to α,β-unsaturated ketones **113**, followed by reaction with sodium acetate, which furnished the α-azido-α,β-unsaturated ketones **114** in good yields [35] (Scheme 48).

**Scheme 49:**
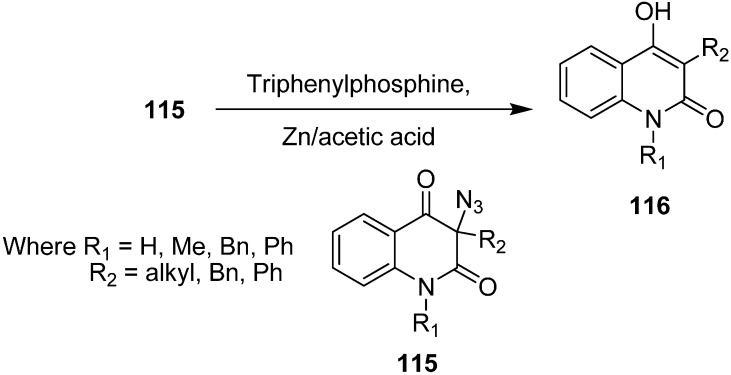
Synthesis of 4-hydroxyquinoline-2(1*H*)-ones **116**.

It has been observed that 3-azidoquinoline-2,4(1*H*,3*H*)-dione reacts in a very different manner from other organic azides, including α-azido carbonyl compounds. For example, Kafka in 2002 [36] explained that during the reduction of azide with triphenylphosphine (Staudinger reaction), the expected 3-aminoquinoline-2,4(1*H*,3*H*)-dione was not formed, but rather de-azidation occured and hence 4-hydroxyquinoline-2(1*H*)-ones **116** were formed. A similar reaction was noticed with 3-azidoquinoline-2,4(1*H*,3*H*)-diones **115** in the presence of acetic acid and zinc (Scheme 49).

The authors investigated the radical chain reactions of azides in addition to their study of the effective reactivity of organic azides under both thermal and photochemical conditions. It was demonstrated that the addition of radical species occured at the α- or γ-position of azido species which after losing a nitrogen molecule were transformed into aminyl radicals.

**Scheme 50:**
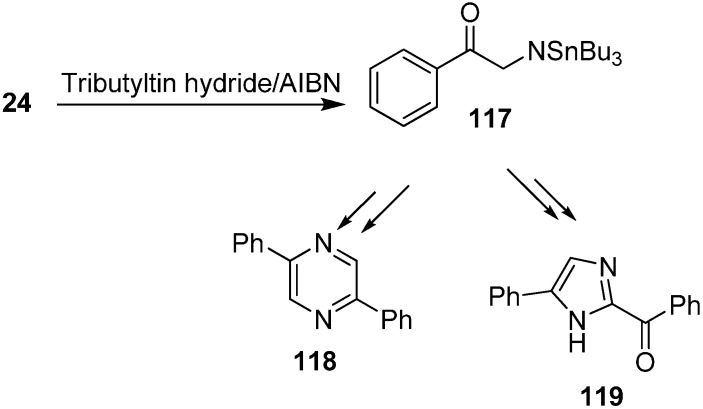
Formation of pyrazine **118** and imidazole **119** from phenacyl azide.

Benati and co-workers [37] reported in 2002 the radical chain reactions of various α-azido ketones such as **24** with tributyltin hydride to form *N-*(tributylstannyl) aminyl radical **117** which might undergo H-abstraction mechanism to form diphenylpyrazine **118** or a 1,2-*H*-shift to form imidazole **119** (Scheme 50).

**Scheme 51:**
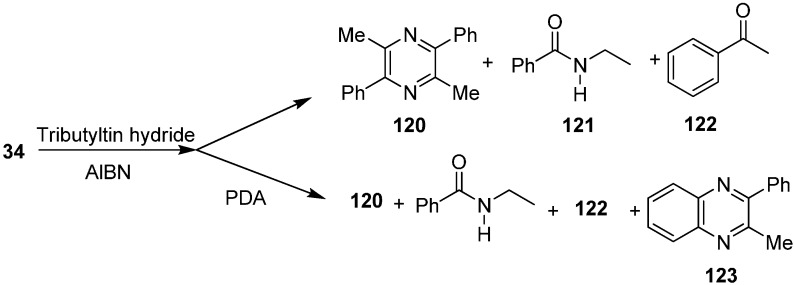
Formation of pyrazine **120** from α-azido ketone **34**.

Their group also reported the reaction of 2-azido-1-phenylpropan-1-one (**34**) with Bu_3_SnH to form pyrazine **120**, nitrogen-inserted benzamide **121** and ketone **122**. They also repeated this reaction in the presence of *o*-phenylenediamine (PDA) and obtained 2-methyl-3-phenylquinoxaline (**123**) (Scheme 51).

**Scheme 52:**
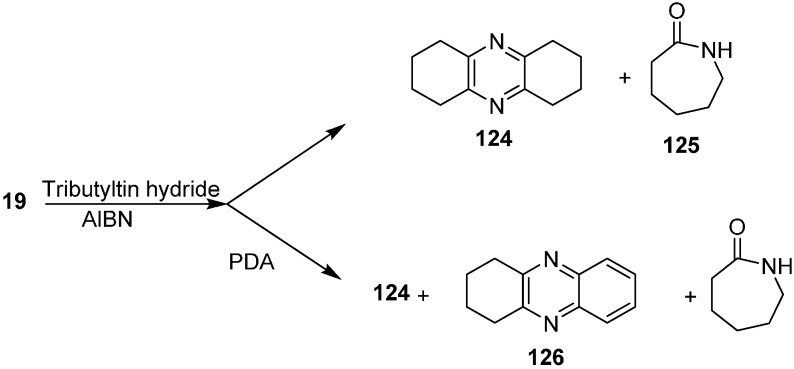
Preparation of pyrazine derivatives **124** and **126** from azido ketone **19**.

The reaction of 2-azidocyclohexanone (**19**) with Bu_3_SnH to form 1,2,3,4,6,7,8,9-octahydrophenazine (**124**) and azepan-2-one (**125**) was also investigated. They repeated this reaction with PDA to obtain 1,2,3,4-tetrahydrophenazine (**126**) (Scheme 52).

**Scheme 53:**
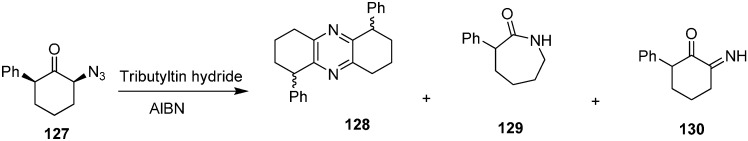
Formation of Compound **128**, **129** and **130** from α-azido ketone **127**.

The reaction of (2*S*,6*S*)-2-azido-6-phenylcyclohexanone (**127**) with Bu_3_SnH formed 1,6-diphenyl-1,2,3,4,6,7,8,9-octahydrophenazine (**128**), 3-phenylazepan-2-one (**129**) and 2-imino-6-phenyl-cyclohexanone (**130**) (Scheme 53).

**Scheme 54:**
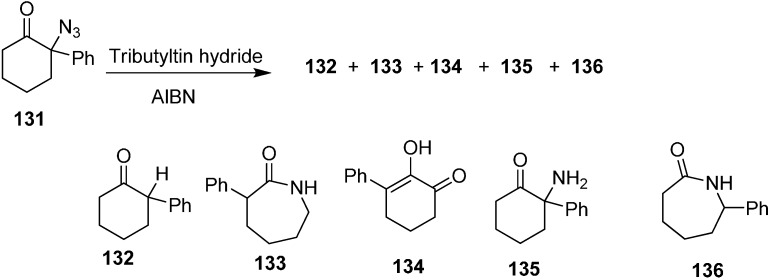
Conversion of azido ketone **131** to products **132**–**136**.

Same group also reported the reaction of 2-azido-2-phenylcyclohexanone (**131**) with Bu_3_SnH to form 2-phenylcyclohexanone (**132**) 3-phenylazepan-2-one (**133**), 2-hydroxy-3-phenylcyclohex-2-enone (**134**), 2-amino-2-phenylcyclohexanone (**135**) and 7-phenylazepan-2-one (**136**) (Scheme 54).

**Scheme 55:**
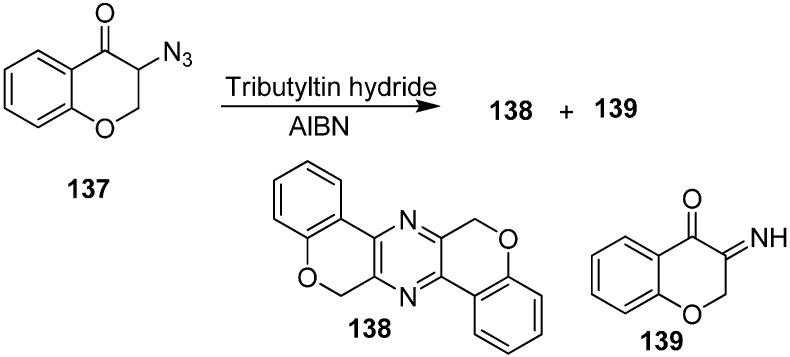
Formation of pyrazine **138** and iminochroman-4-one **139** from **137**.

They also reported the of reaction 3-azidochroman-4-one (**137**) with Bu_3_SnH to form 6,13-dihydrodichromeno[3,4-*b*:3ʹ,4ʹ-*e*]pyrazine **138** and 3-iminochroman-4-one **139** (Scheme 55).

**Scheme 56:**
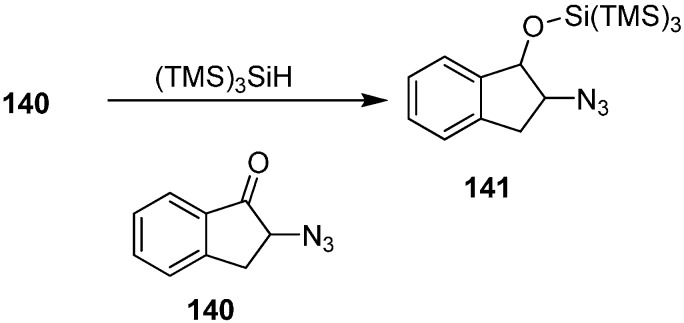
Preparation of silyl ether **141**.

Benati also reported the synthesis of 2-(2-azido-2,3-dihydro-1*H*-inden-1-yloxy)-1,1,1,3,3,3-hexamethyl-2-(trimethylsilyl)trisilane (**141**) from 2-azido-2,3-dihydro-1*H*-inden-1-one **140** by its reaction with (TMS)_3_SiH (Scheme 56).

A wide spectrum of antifungal, antibacterial and antiviral biological activities are associated with the 2-amino-1,3-oxazole moiety. Very recently, BMS-337197 was recognized as powerful inhibitor of inosine monophosphate dehydrogenase (IMPDH). The key structural moiety in BMS-337197 is the central 2-amino-1,3-oxazole moiety.

**Scheme 57:**
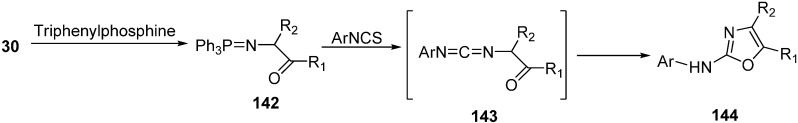
Preparation of 2-(*N*-aryl)-substituted 1,3-oxazoles **144**.

Only few methods are reported for the preparation of 2-(*N*-aryl)-substituted 1,3-oxazoles. A modified preparation of these compounds by using an iminophosphorane/heterocumulene-mediated process was reported by Dhar in 2002 [38]. The utility of 2-(*N*-aryl)-1,3-oxazoles in the synthesis of BMS-337197 was also reported. Rh_2_(OAc)_4_-catalyzed reaction of α-diazoacetophenones with *N*,*N*-disubstituted cyanamides was carried out by Ibata and co-workers and formation of 2-(*N*,*N*-disubstituted)-5-aryl oxazoles was observed. The reaction resulted in the low yield of monosubstituted cyanamides. However, when BMS-337197 (**151**) was synthesized by employing the iminophosphorane/heterocumulene-mediated process, the preparation of 2-(*N*-phenyl)-1,3-oxazoles seemed attractive since the reaction was carried out under mild conditions and gave high yields of the product (Scheme 57).

BMS-337197 was synthesized by using following scheme.

**Scheme 58:**
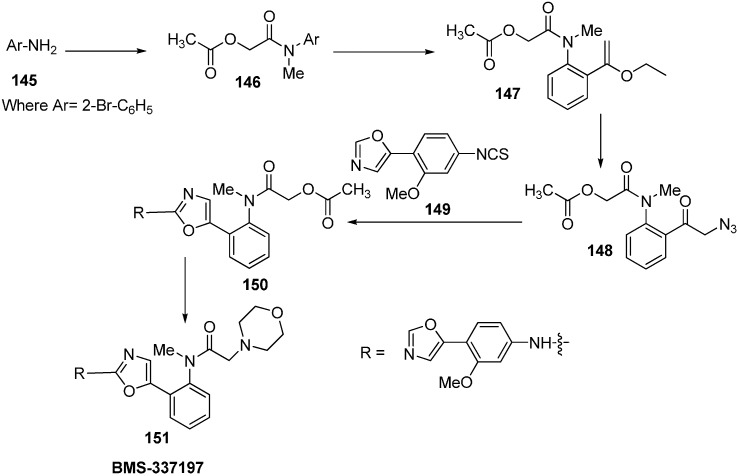
Synthesis of BMS-337197.

Firstly, *o*-bromoaniline (**145**) was treated with acetoxyacetyl chloride followed by methylation to give *N*-methylamide **146**. This *N*-methylamide on heating with tributyl-(1-ethoxyvinyl)tin gave enolether **147**. The enolether on reaction with *N*-bromosuccinamide in water gave a β-ketobromide which upon treatment with sodium azide gave β-ketoazide **148**. The β-ketoazide was then set for iminophosphorane/heterocumulene-mediated annulation. Reaction of the β-ketoazide with isothiocyanate in the presence of CH_2_Cl_2_ at room temperature gave the desired 2-(*N*-aryl)-1,3-oxazole **150** in 20% yield, which was then converted into BMS-337197 (**151**) after two steps as outlined in Scheme 58.

**Scheme 59:**
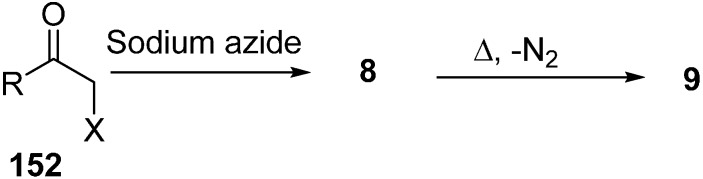
Formation of α-imino ketones **9**.

Erian in 2003 [39] reported in their review paper that α-azido ketone **8** could be synthesized from α-halo ketones **152** by reacting them with NaN_3_ under mild conditions and also described that α-azido ketones could be converted into α-imino ketones **9** by losing N_2_ when heated in an inert solvent (Scheme 59).

**Scheme 60:**
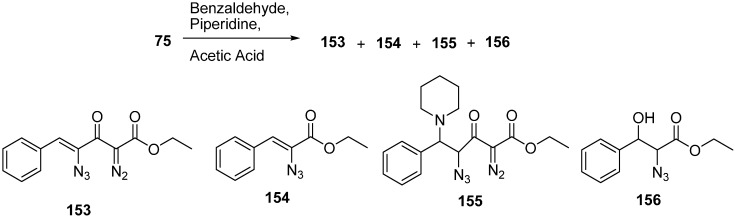
Conversion of ethyl 4-azido-2-diazo-3-oxobutanoate **75** to products **153–156**.

The chemical behaviour of azido diazo esters towards phosphorus (III) reagents was elaborated by Marcus in 2003 [40]. Staudinger reaction with trimethyl phosphite, and chemoselective preparation of diazophosphoramides was also studied. Their research group reported the reaction of ethyl 4-azido-2-diazo-3-oxobutanoate (**75**) with benzaldehyde and piperidine in the presence of acetic acid to form (*Z*)-ethyl 4-azido-2-diazo-3-oxo-5-phenylpent-4-enoate (**153**), (*Z*)-ethyl 2-azido-3-phenylacrylate (**154**), ethyl 4-azido-2-diazo-3-oxo-5-phenyl-5-(piperidin-1-yl)pentanoate (**155**) and ethyl 4-azido-2-diazo-5-hydroxy-3-oxo-5-phenylpentanoate (**156**) (Scheme 60).

**Scheme 61:**
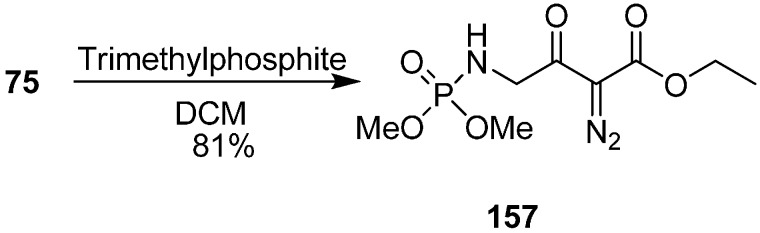
Preparation of γ-(dimethylphosphorylamino)-α-diazo-β-keto esters **157**.

They also reported the reaction of γ-azido-α-diazo-α-keto esters **75** with trimethylphosphite P(OMe)_3_ to form γ-(dimethylphosphorylamino)-α-diazo-β-keto esters **157** by tandem Staudinger/Arbuzov rearrangement under mild conditions with good yield (Scheme 61).

**Scheme 62:**
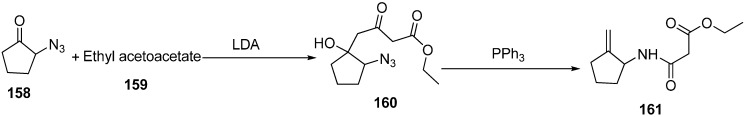
Synthesis of 1-acetamido-2-methylidinecyclopentane **161**.

The synthesis of iminophosphoranes by Staudinger reaction (addition of PPh_3_ to organic azides) is of great synthetic importance. Through hydrolysis of iminophosphoranes, conversion of azides into primary amines is thus permitted. Imines and nitrogen heterocyclic compounds such as pyrroles and pyrrolidines can be synthesized by aza-Wittig reaction of iminophosphoranes with carbonyl compounds. Recently, a new type of PPh_3_-mediated domino procedure *i.e.*, “Staudinger-aza-Wittig-1,5-phosphonium-rearrangement-fragmentation” reaction of 1-azido-2-hydroxy-4,6-dioxohexanes was carried out by Langer in 2003 [41], which resulted in the effective generation of 1-acetamido-2-alkylidinecyclopentanes, amides and lactams. These products are very useful precursor in organic synthesis and are of great pharmacological relevance. In this scheme, the dianion of ethyl acetoacetate (**159)** was reacted with α-azidocyclopentanone **158** to form 1-azido-2-hydroxycyclopentane **160**, followed by treatment with triphenylphosphine to form 1-acetamido-2-methylidinecyclopentane **161** in 60% yield (Scheme 62).

In order to expand the scope of greener organic reaction methods, [hydroxy(2,4-dinitrobenzene sulfonyloxy)iodo]benzene (HDNIB)- mediated effective synthesis of α-azido ketones and α-thiocyanoketones was done by Lee in 2005 [42]. This reaction was carried out in ionic liquids such as 1-butyl-3-methylimidazolium tetrafluoroborate, [bmim]BF_4_. Originally, the probability of α-sulfonyoxylation reaction of ketones was tested by using HDNIB in [bmim]BF_4_.

**Scheme 63:**
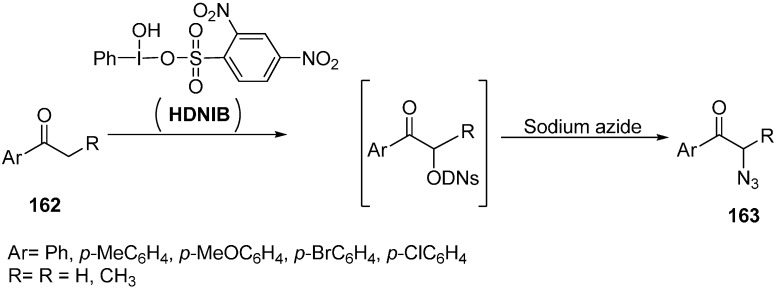
Preparation of α-azido ketone **163** using HDNIB.

The traditional synthesis of α-organosulfonyloxy ketones had been mostly carried out in organic solvents such as acetonitrile and dichloromethane. It was demonstrated that hypervalent iodine (III) sulfonates were incompatible with highly polar reaction media such as methanol and water. Therefore, it was desired to produce a synthetic protocol which could be carried out in highly polar media. Ionic liquida was thus used for expanding the synthetic uses of hypervalent iodine (III) sulfonates. In this reaction procedure, aryl ketones **162** were allowed to react with HDNIB in [bmim]BF_4_ which resulted in the successful formation of α-[(2,4-dinitrobenzene)-sulfonyl]oxy ketone intermediate in high yields. After determining suitable conditions for successful α-sulfonyloxylation of ketones in ionic liquid, the α-azidation of ketone was accomplished by reacting sodium azide with the α-[(2,4-dinitro-benzene)sulfonyl]oxy ketone intermediate (Scheme 63).

Both aromatic methyl ketones and methylene ketones provided α-azido ketones in high yields. Then replacement of α-[(2,4-dinitrobenzene)sulfonyl]oxy ketone intermediate was done with thiocyanate ion which resulted in α-thiocyanato ketones.

2-Alkylidenepyrrolidines are very useful building blocks that have displayed noticeable applications in organic synthesis and are valuable synthetic predecessors of pyrroles. The pyrrole ring appears in a large number of pharmaceutically active natural compounds and wonder drugs (e.g., zomepirac and atorvastatin). Keeping in view the synthetic utility of pyrroles, the condensation reaction between α-azido ketones and 1,3-dicarbonyl dianions was carried out by Freifeld in 2006 [43]. This reaction procedure resulted in the open chain condensed products that were transformed into pyrroles by Staudinger-Aza-Wittig reactions followed by subsequent treatment with trifluoroacetic acid (TFA).

**Scheme 64:**
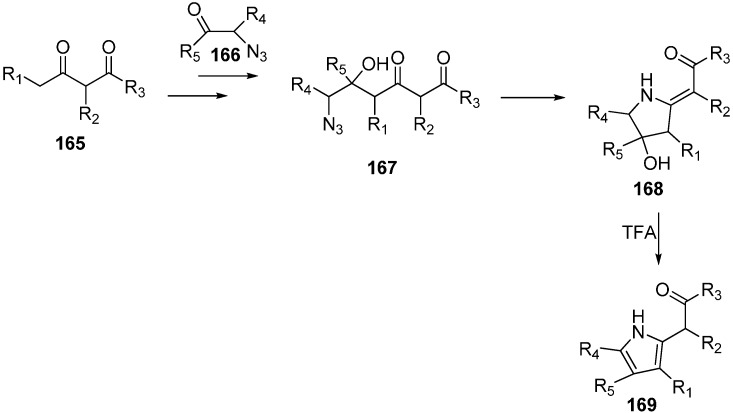
Synthesis of pyrroles **169**.

In this reaction procedure, the reaction of dianions of 1,3-dicarbonyl compounds **165** with α-azido ketones **166** gave 6-azido-5-hydroxy-3-oxoalkanoates **167**. The latter then resulted in 2-alkylidenepyrrolidines **168** by intramolecular Staudinger-aza-Wittig reaction. 2-Alkylidene-pyrrolidines were then transformed into the pyrroles **169** by reacting with trifluoroacetic acid (TFA) (Scheme 64).

**Scheme 65:**
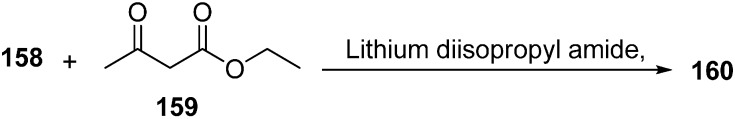
Reaction of ethyl acetoacetate **159** with α-azido cyclopentanoate **158**.

It is known that iminophosphorane formation has been extensively used for the synthesis of primary amines, imines and different nitrogen heterocycles since iminophosphoranes are readily available by the reaction of triphenylphosphine (PPh_3_) with azides in the Staudinger reaction. Rather than normal aza-Wittig reactions, new cyclization reactions of different dianions were discovered in which PPh_3_- mediated conversion of γ-azido-β-hydroxyketone was carried out by Freifeld in 2006 [44]. In this transformation, condensation of 1,3-dicarbonyl dianions with 2-azidocyclopentanone was accomplished, which resulted in the formation of 2-azido-1-hydroxy-1-(2,4-dioxoalkyl)cyclopentanes which was followed by treatment with PPh_3_ to form 1-(1,3-dioxoalkyl)amino-2-alkyl-(alkylidine)cyclopentanes by means of a domino “Staudinger/semi-aza-Wittig/fragmentation” reaction. For example, by reacting the dianion of ethyl acetoacetate (**159**) with α-azido cyclopentanoate (**158**), 2-azido-1-hydroxy-1-(4-ethoxy-2,4-dioxobutyl)cyclopentane (**160**) was formed in good to low yield (Scheme 65).

**Scheme 66:**
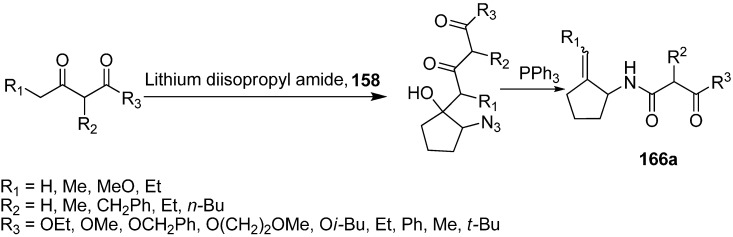
Conversion of azido ketone **158** to compound **166a**.

Similarly, the reaction of various 1,3-dicarbonyl dianions of methyl, benzyl, methoxyethyl and *iso*-butyl acetoacetate with α-azido cyclopentanone was studied, which resulted in the formation of 2-azido-1-hydroxy-1-(2,4-dioxoalkyl) cyclopentanes, followed by treatment of the latter with PPh_3_ to form 1-(4-alkoxy-2,4-dioxobutyl)amino-2-(methylidine)cyclopentanes **166a** (Scheme 66).

**Scheme 67:**
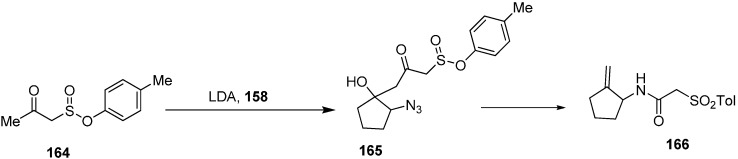
Synthesis of derivatives **166** from azido ketone **158**.

Same group performed the condensation of the dianion of tosylacetone (**164**) with α-azidocyclopentanone (**158**) which resulted in 2-azido-1-hydroxy-1-(3-tosyl-2-oxopropyl)cyclopentane (**165**) in high diastereoselectivity. The reaction of later with PPh_3_ resulted in the formation of 1-amino-2-(methylidine)cyclopentane (**166**) (Scheme 67).

**Scheme 68:**
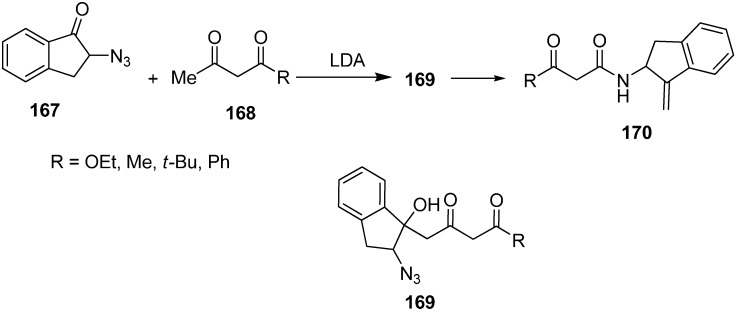
Preparation of compound **170** from azido ketone **167**.

The reaction of 2-azidoindane-1-one **167** with dianion of ethylacetoacetate **168** gave 2-azido-1-hydroxy indane **169** followed by treatment with PPh_3_ to form 2-amino-1-(methylidine)indane **170** (Scheme 68).

**Scheme 69:**
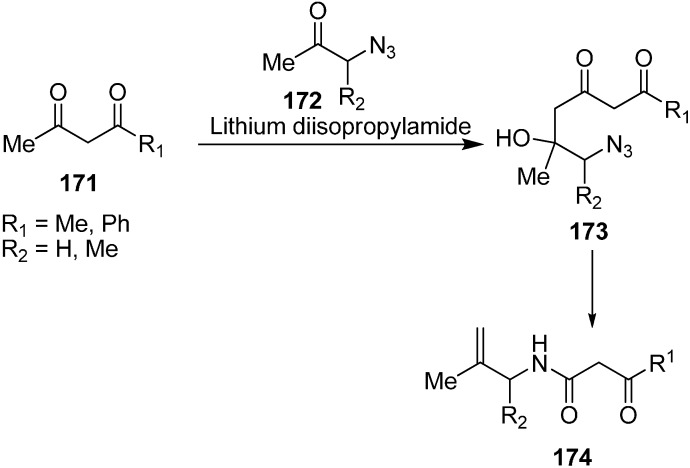
Conversion of azido ketone **172** to compound **174**.

They also studied the reaction of 1,3-dicarbonyl dianions **171** with straight chain azides **172** as shown below (Scheme 69).

**Scheme 70:**
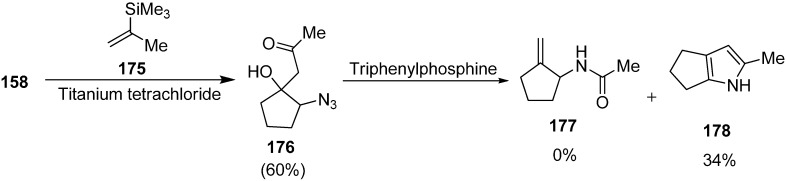
Preparation of 2-azidocyclopentanone **158**.

Moreover, the synthesis of 1-(2-azido-1-hydroxycyclopentyl) propan-2-one (**176**) in 60% yield by reaction of trimethyl (prop-1-en-2-yl) silane (**175**) and 2-azidocyclopentanone (**158**) in the presence of TiCl_4_ and CH_2_Cl_2_ was also reported by Freifeld (Scheme 70).

The use of organohypervalent iodine reagents is a very fertile and very attractive field in organic synthesis as various hypervalent iodine reagents, iodobenzene diacetate (IBD) and [hydroxy(tosyloxy)-iodo]benzene (HTIB) (Koser’s reagent) are found to be more useful than other reagents such as iodosobenzene (IOB), *etc.* IOB is found to be less useful because it is polymeric in nature and hence insoluble in common solvents. To solve these problems, combination reagents were introduced. For example, the effectivity of IOB was greatly enhanced by its combination with acids, bases or salts.

**Scheme 71:**
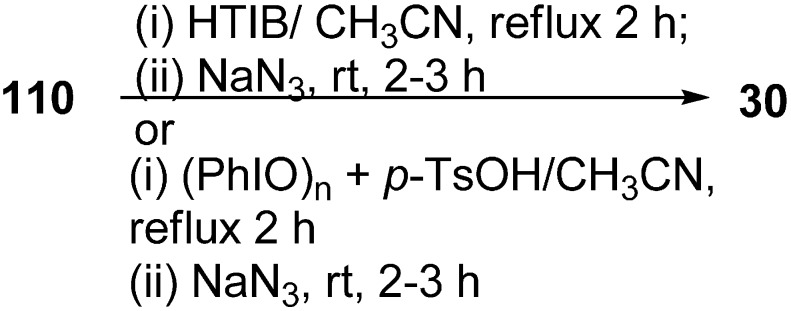
Synthesis of α-azido ketones.

Prakash in 2006 reported the reaction of various ketones with [hydroxy(tosyloxy)iodo]benzene (HTIB), followed by subsequent treatment of α-tosyloxy ketones (generated *in situ*) with NaN_3,_ for the generation of α-azido ketones [45]. HTIB, which was used in this reaction, was synthesized by using iodosobenzene combined with *p*-toluenesulphonic acid. In this reaction, firstly oxidation of ketone **110** was done with HTIB (one equiv.) in acetonitrile as a solvent which caused the generation of α-tosyloxy ketone **79** *in situ*, and the resulting α-tosyloxy ketone was then treated with NaN_3_. The reaction resulted in the formation of corresponding α-azido ketones **30** in good yields (Scheme 71).

In another advancement, in place of HTIB, iodosobenzene and *p*-toluenesulphonic acid [(PhIO)_n_+*p*-TsOH] were used in combination, which produced *in situ* HTIB, which reacted with ketone **110** and gave intermediate α-tosyloxy ketones **79**.

**Scheme 72:**
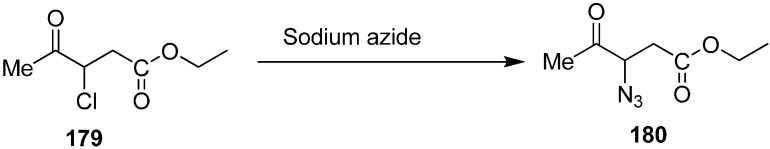
Preparation of ethyl 3-azido-4-oxopentanoate **180**.

α-Enamino esters, as efficient building blocks in organic synthesis, are important precursors for biologically active compounds such as β-amino acids and heterocyclic compounds. For this reason, the preparation of γ-imino-β-enamino esters was reported by Mangelinckx in 2006 [46] by condensing primary alkylamines with 3-azido-4-oxopentanoate. Initially, nucleophilic displacement of ethyl 3-bromo-4-oxopentanoate with azide in acetone in the presence of triethylamine was carried out. The β-azido ester **180** could only be separated after limited heating than at prolonged heating, since prolonged heating caused removal of molecular nitrogen and resulted in the formation of ethyl 3-amino-4-oxo-2-pentanoate. For this reason, a more efficient method was reported for the preparation of of ethyl 3-azido-4-oxopentanoate from easily accessible ethyl 3-chloro-4-oxopentanoate (**179**) in the presence of excess sodium azide in acetone under refluxing conditions (Scheme 72).

**Scheme 73:**
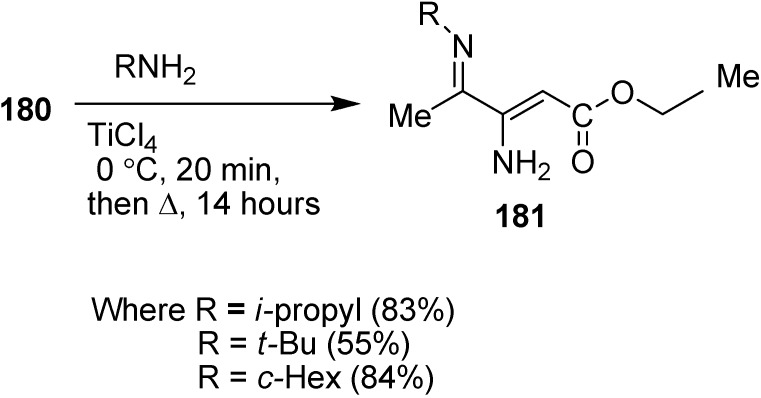
Synthesis of 4-alkylimino-3-amino-2-pentenoates **181**.

In early times, it was demonstrated that after condensing unfunctionalized α-azido ketones with primary amines, a mixture of α-diimines and α-azido ketimines were formed, whose preparation depended upon reaction conditions and steric hindrance in the substrate but in contrast to these results, ethyl 3-azido-4-oxopentanoate (**180**) on reaction with primary amines in the presence of titanium (IV) chloride gave 4-alkylimino-3-amino-2-pentenoates **181** as single stereoisomers of undefined *E*/*Z* stereochemistry in 55%–84% yield (Scheme 73).

Inosine monophosphate dehydrogenase (IMPDH) is an important enzyme that causes the nicotinamide adenosine dinucleotide (NAD)-catalyzed conversion of inosine 5ʹ-monophosphate (IMP) to xanthosine 5ʹ-monophosphate (XMP). IMPDH is an appealing goal for anticancer and antiviral therapies. For the purpose to recognize novel IMPDH inhibitors, *N*-(2-(2-(3-methoxy-4-(oxazole-5-yl)phenylamino)oxazol-5-yl)phenyl)-*N*-methyl-2-morpholinoacetamide(BMS-337197) was discovered.

**Scheme 74:**
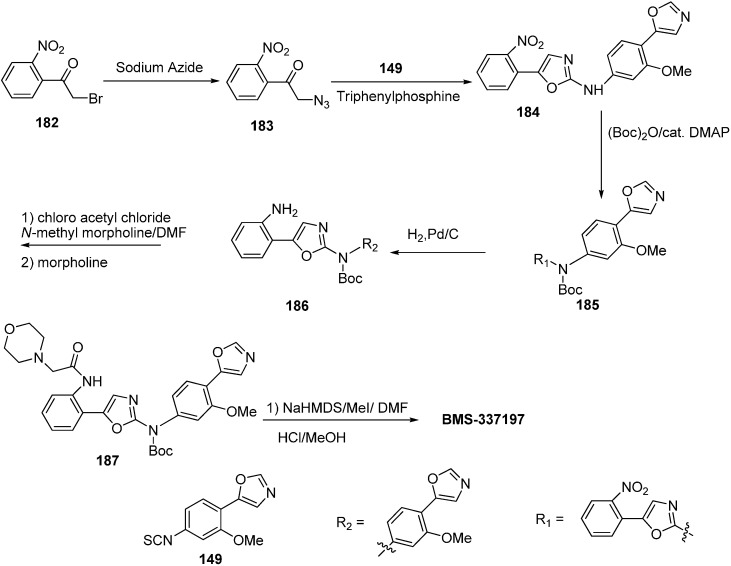
Synthesis of BMS-337197.

To synthesize BMS-337197 on large scale, a new and efficient pathway was reported by Zhao in 2007 [6]. In this approach, 2-bromo-1-(2-nitrophenyl)ethanone **182** was used for the synthesis of α-azido ketone, acting as an intermediate for BMS-337197 synthesis. BMS-337197 was synthesized with overall yield of 55% on multigram scale (Scheme 74).

**Scheme 75:**
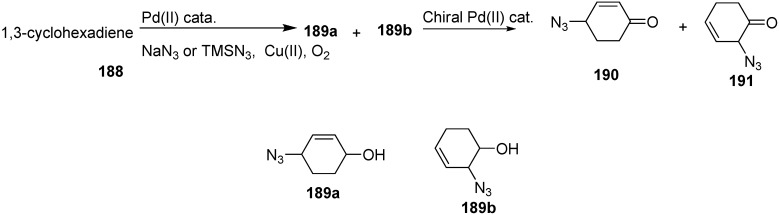
Synthesis of azido ketones **190** and **191** from 1,3-cyclohexadiene **188**.

A new asymmetric synthetic procedure for the addition of azide ion to 1,3-dienes to produce chiral azido-substituted ketones was developed by El-Qisairi in 2008 [47]. In this approach, the Pd(II)-catalyzed synthesis of α-azido ketones from 1,3-cyclohexadiene (**188**) was revised. The reaction involved the oxidation of 1,3-cyclohexadiene in the presence of azide ions and mixed aqueous solvents. As a result of this reaction 4-azido-2-cyclohexene-1-one (**190**) and 2-azido-3-cyclohexene-1-one (**191**) was formed at about 70% yield. The reaction was net air oxidation and the conversion of compound **190** to compound **191** involving [3,3] sigmatropic rearrangement. The overall reaction is summarized in Scheme 75.

Many α-azido, α-bromo and α-nitro ketones are species that undergo asymmetric reduction by using lyophilized cells of easily available *Comamonas testosteroni* DSM 1455, which showed excellent catalytic activity for asymmetric reduction. This reaction procedure, proposed by Wallner in 2008 [48], occured at moderate substrate concentration (16 g/L) in a ‘substrate-coupled’ approach. Here 20% (*v*/*v*) of 2-propanol was used as hydrogen donor. Also naturally less common anti-Prelog stereoselectivity was achieved. Different metal complexes and biocatalytic processes were used for the stereoselective reduction of ketones to their respective enantiopure alcohols **195**. Many microbial strains contain alcohol dehydrogenases (ADHs) which catalyze the stereoselective transfer of hydride from the cofactor, NAD(P)H, to Si- or Re- face of the carbonyl group and result in the synthesis of corresponding (*S*)- or (*R*)-alcohols.

**Scheme 76:**
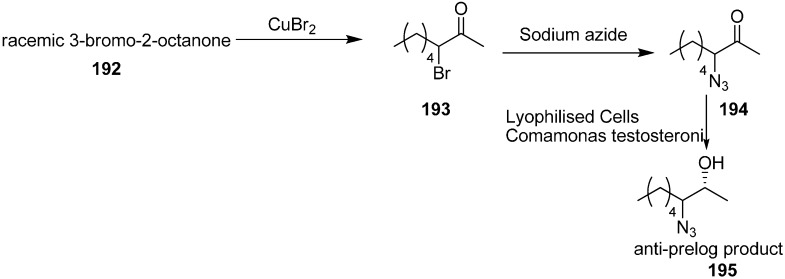
Preparation of azido ketone **194** and azido alcohol **195**.

By using the method of King and Ostrum, racemic 3-bromo-2-octanone (**193**) was obtained. In this approach, CuBr_2_ was used, which after reduction to CuBr, coordinated to the enolate ion. By employing the reaction conditions, the thermodynamic product (addition in position 3) was favoured over the kinetic product (addition in position 1). Racemic 3-azido-2-octanone (**194**) was obtained in excellent yield by reacting **193** with sodium azide in the mixture of acetic acid and ethanol at low temperature (Scheme 76).

1,3-Dialkylimidazolium cations based ionic liquids are of considerable importance and have been utilized as green substituent to volatile organic solvents in recent years. Due to their volatile nature, the use of ionic liquids causes the minimum solvent utilization and addresses the problem of volatile organic solvent (VOS) emissions to the environment. Due to this reason, ionic liquids are termed as envoirnmentally fascinating substituents to the traditional organic solvents.

**Scheme 77:**
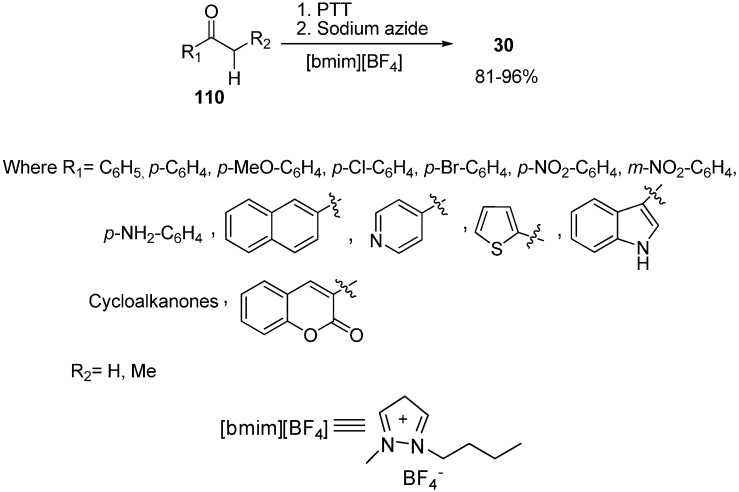
Synthesis of α-azido ketones.

In extension to the use of ionic liquids as another reaction medium for different organic conversions, a simple and effective one pot synthesis of α-azido ketones from various ketones was reported by Kumar in 2009 [49]. In this reaction scheme, different ketones (cyclic, acyclic, aromatic, heterocyclic) having α-hydrogen were made to react with phenyltrimethylammonium tribromide (PTT), followed by reacting with NaN_3_ in ionic liquid and as a result α-azido ketones were synthesized in high yields (Scheme 77).

**Scheme 78:**
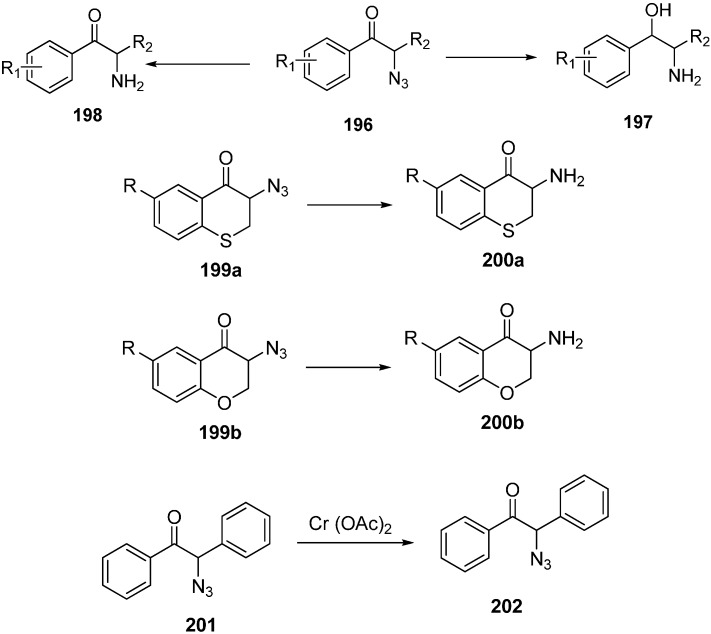
Reduction of azido ketones.

The reduction of α-azido ketones to α-amino ketones using tin (II) chloride followed by Boc group protection was reported by Patonay in 2009 [50]. Cr (II) was also used as catalyst for this reduction, but the products were formed in low yields (Scheme 78).

**Scheme 79:**
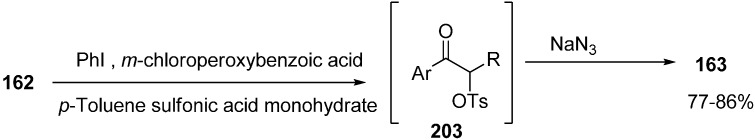
Preparation of α-azido ketone **163**.

α-Azido ketones can be commonly synthesized by azide-halogen exchange reaction of α-halo ketones and also by the reaction of sodium azide with α-tosyloxy ketones. Recently iodobenzene-catalyzed synthesis of α-azido ketones and α-thiocyano ketones was achieved by Chang [51]. The method involved the generation of of α-azido ketones and α-thiocyano ketones from aryl ketones without the isolation of α-tosyloxy ketones in good to the excellent yields (Scheme 79).

**Scheme 80:**
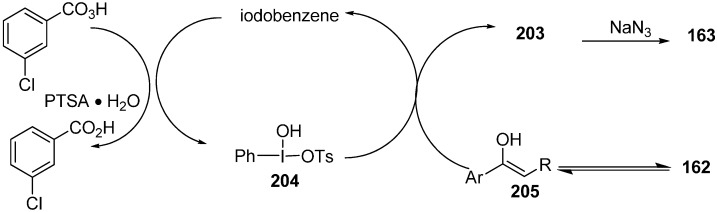
Pathway for α-azidation.

Moreover, the nucleophilic substitution reaction of α-tosyloxy ketone intermediates with thiocyanate ion was also observed, which gave the corresponding α-thiocyano ketones with equal efficiency to that of α-azidation reactions. A credible path for the procedure is shown in Scheme 80.

Firstly, the PhI was oxidized with MCPBA in the presence of PTSA to produce [hydroxyl(tosyloxy)iodo]benzene **204** *in situ* which then produced α-tosyloxyketone intermediates **203** by the reaction with the enol form **205** of the ketone. Nucleophilic substitution of **203** with azide ion gave the required α-azido ketones **163**. The enhanced rate of the reaction, mild reaction conditions, high yield, avoidance of toxic catalysts and reuse of the reaction medium were the most important aspects of this reaction procedure.

**Scheme 81:**
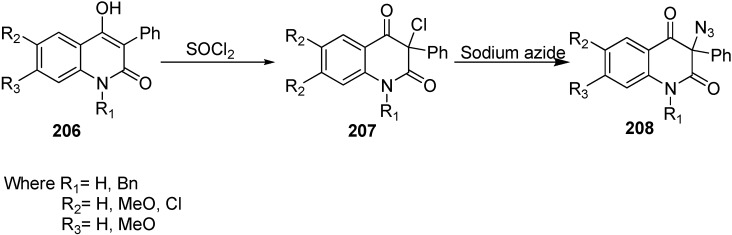
Preparation of 3-azidoquinoline-2,4(1*H*,3*H*)-diones **208**.

Moreover, the synthesis of 3-azidoquinoline-2,4(1*H*,3*H*)-diones **208** was achieved by Kafka in 2011 [36] from readily accessible 4-hydroxyquinoline-2-(1*H*)-ones **206**. 4-Hydroxyquinoline-2-(1*H*)-ones were halogenated (chlorinated/brominated) with sulfuryl chloride and bromide to form the corresponding 3-chloroquinoline-2,4(1*H*,3*H*)-diones **207** and 3-bromoquinoline-2,4(1*H*,3*H*)-diones. Nucleophilic substitution of 3-halogenoquinoline-2,4(1*H*,3*H*)-diones with sodium azide resulted in the direct synthesis of 3-azidoquinoline-2,4(1*H*,3*H*)-diones **208** in quantitative yield (Scheme 81).

**Scheme 82:**
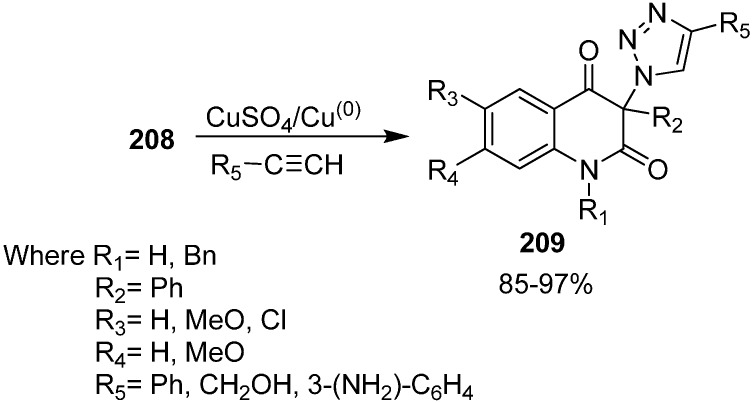
[3+2] cycloaddition of 3-azidoquinoline-2,4(1*H*,3*H*)-diones **208**.

The reactivity of 3-azidoquinoline-2,4(1*H*,3*H*)-diones **208** was also tested by reaction with terminal acetylenes such as phenylacetylene, propargyl alcohol and 3-ethnylaniline using copper (I) catalyst for [3+2] cycloaddition (Scheme 82).

Initially, copper (II) sulphate pentahydrate and ascorbic acid were tested as a source of copper(I) while *tert*-BuOH/H_2_O was used as a solvent. Similarly, a combination of copper(II) acetate and elemental copper in acetonitrile was also used, but these reactions failed due to the low solubility of azides in these reaction media. However, when the reaction was carried out by combining copper(II) sulphate pentahydrate and elemental copper, the obtained results were better than with previous methodology. Carrying out the cycloaddition between azides and acetylene in DMSO in the presence of copper(II) sulphate pentahydrate and elemental copper, the required 1,4-disubstituted 1,2,3-triazoles **209** were formed in moderate to excellent yields.

**Scheme 83:**
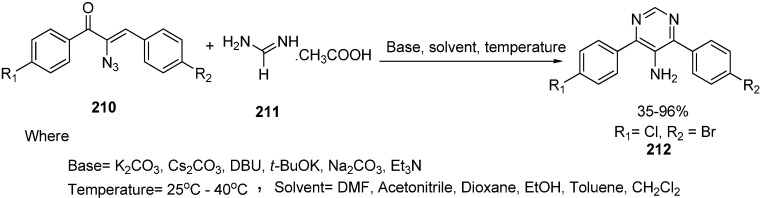
Preparation of pyrimidine derivative **212** from azido ketone **210**.

5-Aminopyrimidines show various pharmacological activities such as antianoxic and antilipid peroxidation activities. Polysubstituted pyrimidines have been prepared using different procedures based on condensation reactions between *C*-*C*-*C* and *N*-*C*-*N* or cross-coupling reactions. Reduction of 5-nitropyrimidine to 5-aminopyrimidine is most common method employed so far. Synthesis of polysubstituted 5-aminopyrimidines **212** using α-azido vinyl ketones **210** and amidines **211** was carried out by Hu in 2011 [4]. The reaction was performed using mild basic conditions with excellent yield. Also the 1,4-Michael addition mechanism of this synthesis was reported (Scheme 83).

**Scheme 84:**
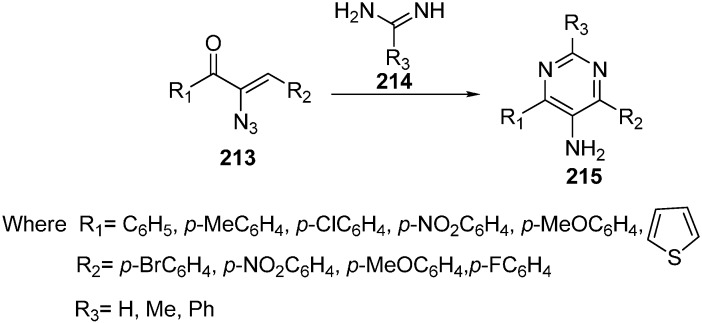
Synthesis of 5-amino pyrimidines **215**.

With suitable reaction conditions, the reaction was observed for a variety of α-azido vinyl ketones and amidines (Scheme 84).

**Scheme 85:**
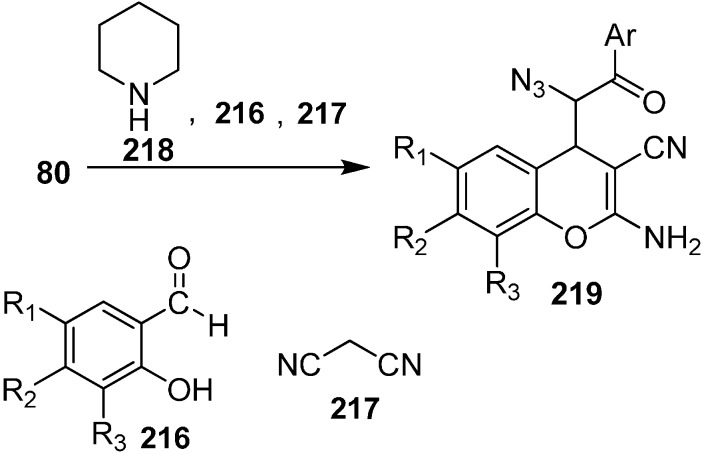
Synthesis of azido chromene **219**.

An efficient and simple synthesis of highly functionalized azido chromene derivatives was reported by Babu in 2011 [52]. 2-Aminochromenes are used in many naturally occurring products and in pigments, cosmetics, and agrochemicals. In these three component reactions, salicylaldehyde, malononitrile and many other nucleophiles such as phenacyl azides were involved. In this approach, phenacyl azide was added to the iminocoumarin derivative, which was obtained by the reaction of salicylaldehyde **216** and malononitrile **217** in ethanol at room temperature in the presence of an equivalent quantity of piperidine. The reaction was complete in 30 min and hence azido chromene **219** was formed with overall yield of 67% as a mixture of *syn* and *anti* isomers. However, when the reaction was carried out by using a catalytic amount of piperidine, the reaction took 55 min and resulted in an 85% overall yield of the product **219** (Scheme 85).

Various salicylaldehydes having either electron-withdrawing substituents (such as halide) or electron-donating substituents (such as alkoxy groups) gave the required products in high yield under similar conditions. It was observed that 1,6-substitited azido chromenes preferably displayed *syn* selectivity while in case of 8-substituted azido chromenes, *anti* diastereoselectivity was observed.

Nitrogen-containing heterocycles, also known as azaheterocycles, occur in a variety of natural and biologically active products. For their formation, click reactiona or more effectively Huisgen [3+2] cycloaddition has been originated as a ‘near perfect’ *C-N* bond forming reaction for the preparation of *N*-substituted 1,2,3-triazoles. This reaction is easily accelerated in the presence of Cu(I) catalyst and results in the formation of the 1,4-disubstituted isomer.

**Scheme 86:**
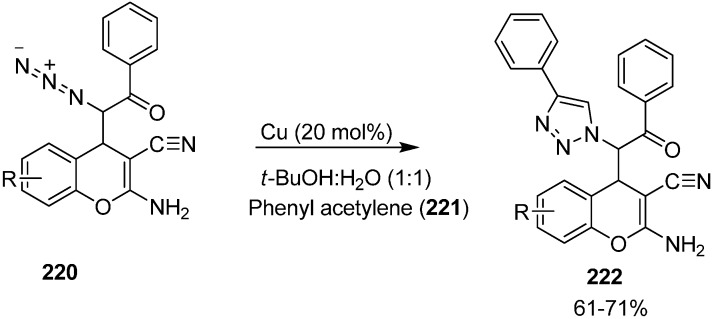
Click reaction of α-azido chromenes **220**.

In continuation of work in this area, the click reaction has also been applied to these α-azido chromenes and hence heterocyclic structures resulted by attempting [3+2] cycloaddition reaction between phenylacetylene and azidochromenes. In this case, the *syn* isomer of azido chromene **220** was treated with phenylacetylene **221** in 1:1 mixture of *t*-BuOH and H_2_O in the presence of CuI as a catalyst which resulted in the formation of expected 1,4-disubstituted 1,2,3-triazole derivative **222** in 71% yield (Scheme 86).

**Scheme 87:**
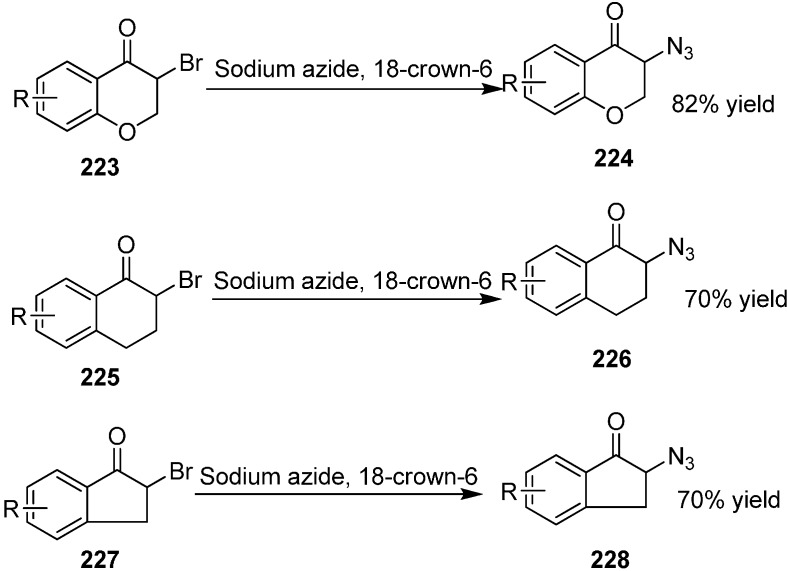
Synthesis of chiral cyclic α-azido ketones.

Chiral cyclic α-azido ketones are very useful and important substrates in organic chemistry because they reduce to form biologically active compounds such as amino alcohols. α-Azido derivatives of tetralone, indanone, chromanone and thiochromanone structures are very important due to their enhanced biological activities originating from their cyclic structures. Firstly, these α-azido ketones were synthesized by Canbolat in 2012 [53]. They synthesized 15 different types of racemic α-azido ketones and in these reactions NaN_3_ was used as azide source and crown ether was used as phase transfer catalyst. The yield of the reactions ranged from 60%–80%. These compounds were analyzed by HPLC with a chiral coloumn (Scheme 87).

**Scheme 88:**
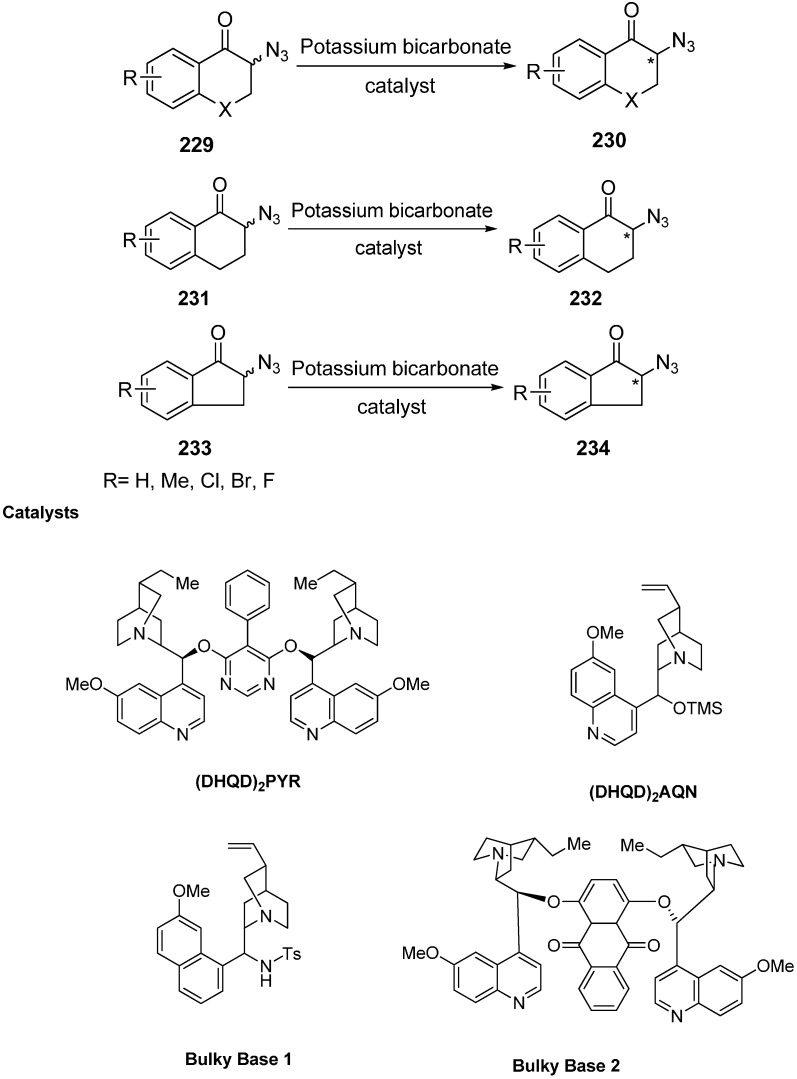
Deracemization of chiral cyclic α-azido ketones.

Moreover, deracemization of numerous α-azido ketone derivatives was carried out in the presence of cinchona alkaloids and hence resulted in the formation of chiral α-azido ketones (α-azido chromanone, tetralone and indanone derivatives) in one step. To carry out these reactions, four different kinds of cinchona alkaloids were selected. The heterogeneous mixture of α-azido ketone, the catalyst and KHCO_3_ was stirred at room temperature for deracemization (Scheme 88).

**Scheme 89:**
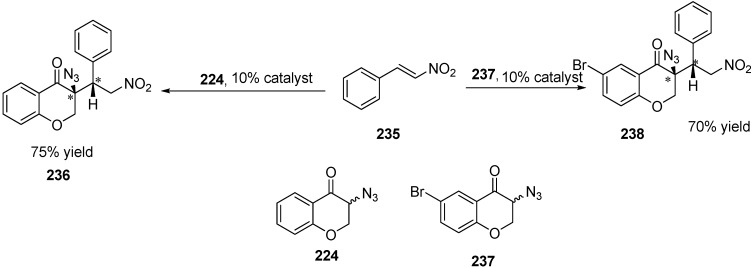
Michael addition of β-nitrostyrene to azidochroman derivatives.

In addition to deracemiation reactions, some other reactions were also performed. For example, Michael addition of two α-azido ketones (3-azidochroman-4-one (**224**) and 3-azido-6-bromochroman-4-one (**237**)) to β-nitrostyrene (**235)** was also done to obtain two chiral centres (Scheme 89).

**Scheme 90:**
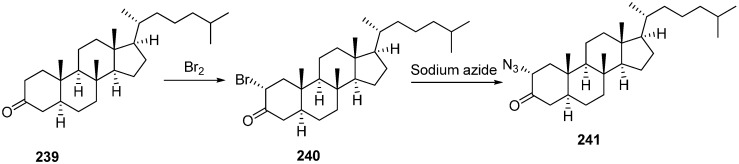
Synthesis of 2α-azido-5α-cholestan-3-one (**241**).

In extension of the work to synthesize steroidal heterocycles, a very simple and efficient pathway was developed by Kadar in 2012 [3] to form novel 2α-triazolyl cholestane derivatives by using α-azido ketones. In this approach, novel steroid derivatives were synthesized *via* copper(I)-catalyzed azide alkyne cycloaddition (CuAAC) reaction. Here, 2α-azido-5α-cholestan-3-one (**241**) was selected as starting material. 2α-Bromo-5α-cholestan-3-one (**240**) was obtained from readily accessible cholestanone **239** by bromination After separating the α-bromo ketone, the compound was stirred for 8 h in the presence of NaN_3_ to synthesize the required 2α-azido ketone **241** in good yield (Scheme 90).

**Scheme 91:**
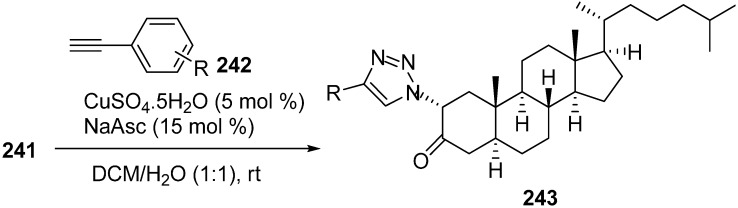
Dipolar cycloaddition with terminal alkynes.

The synthesized 2α-azido-5α-cholestan-3-one (**241**) was then converted to several A-ring-substituted 1,2,3-triazolylcholestan-3-ones **243** in good yield by reaction of **241** with various terminal alkynes **242** (Scheme 91).

**Scheme 92:**
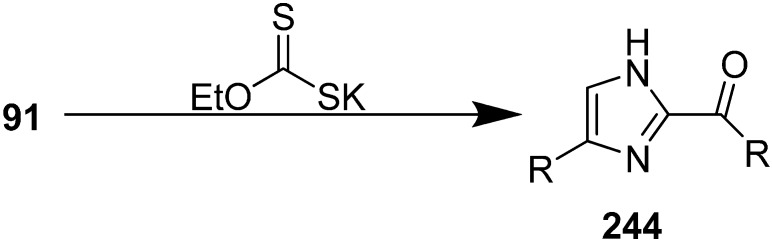
Synthesis of disubstituted imidazole derivatives **244**.

Harschneck [54] reported in 2012 a convenient and practical approach for the synthesis of α-azido carbonyls from 1,3-dicarbonyls. Their reaction methodology involved the use of NaN_3_ and an iodide- based oxidizing agent (I_2_ or IBX-SO_3_K/NaI). They could achieve high yields of α-azido ketones as a result. Kamble in 2012 [55] developed a methodology for the preparation of α-azido ketones using NaIO_4_-NaN_3_ and generally high yields were achieved. Imidazole is a very useful heterocyclic moiety, used in the generation of *N*-heterocyclic carbenes. This moiety exists in some important molecules such as biotin, histindine, topsentine, nortopsentine and also shows a wide variety of biological activities. Chen in 2013 reported an effective, one-pot synthesis of new imidazole derivatives from α-azido ketones in the presence of potassium ethyl xanthate [56]. In this procedure, potassium ethyl xanthate was used as catalyst and the disubstituted imidazole **244** derivative was synthesized *via* dimerization of an arylglyoxal imino derivative generated from α-azido keotones **91** (Scheme 92).

**Scheme 93:**
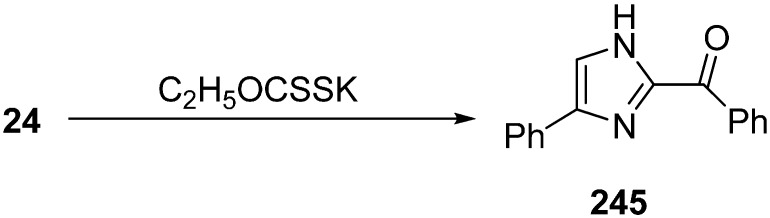
Synthesis of imidazole **245** from α-azido ketone **24**.

The reaction of 2-azido-1-phenylethanone was selected as a model reaction. In the absence of base, no reaction occurred. After testing different bases, it was demonstrated that potassium ethyl xanthate was the most efficient one. Among various protic and aprotic solvents, *i-*PrOH was the most effective solvent for the optimization of reaction conditions. The reaction was also observed at different temperatures and at different amount of bases. The yield was low at lower temperature or by using lower quantity of additives. On the basis of initial study, efficient reactivity was achieved in *i-*PrOH at 70 °C when potassium ethyl xanthate (0.5 equiv.) was used (Scheme 93).

**Scheme 94:**
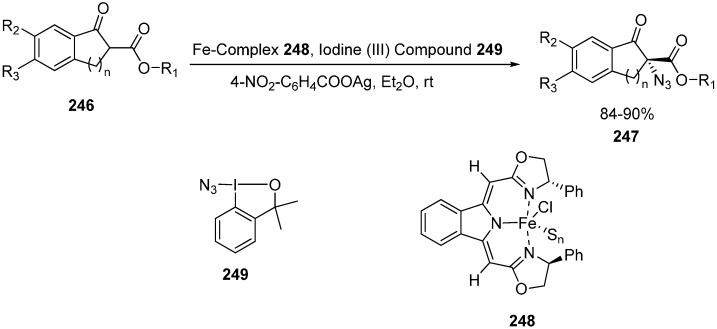
Iron-catalyzed α-azidation of β-keto esters.

Deng in 2013 reported the first example involving the use of Fe-catalyzed α-azidation of β-keto esters. The methodology was applied to a variety of β-keto esters and oxindoles and high enantioselectivities were obtained in the products (up to 93% ee and 94% ee, respectively) [57] (Scheme 94).

Vita in 2013 employed azidobenziodoxole for the azidation of β-keto esters and excellent yields (ranging from 79%‒quantitative) were obtained [58]. The methodology was also applied to silyl enol ethers as well for the synthesis of α-azido ketones, and moderate to good yields could be obtained.

## 3. Conclusions

In summary, the synthesis of α-azido ketones can be achieved by a number of pathways. Further, synthetic importance of α-azido ketones has been clearly demonstrated by their numerous reactions covered in this review article.
